# Phosphoserine as an Alternative Energy Source for *E. coli* Cell-Free Protein Synthesis with Increased Yield and Prolonged Activity

**DOI:** 10.1021/acssynbio.5c00649

**Published:** 2026-06-18

**Authors:** Shanny Ackerman, Yael Fink, Yasmin Habib, Devora Cohen-Karni, Omer Adir, Gal Chen, Yuval Richtman, Sónia Siquenique, Michael Levi, Jeny Shklover, Bruno Sarmento, Avi Schroeder

**Affiliations:** † The Louis Family Laboratory for Targeted Drug Delivery and Personalized Medicine Technologies, Department of Chemical Engineering, 26747 Technion − Israel Institute of Technology, Haifa 32000, Israel; ‡ College of Osteopathic Medicine, Lake Erie College of Osteopathic Medicine, Greensburg, Pennsylvania 15601, United States; § Department of Biological Engineering, MIT, Cambridge, Massachusetts 02139, United States; ∥ i3S − Instituto de Investigação e Inovação em Saúde, 451168 Universidade do Porto, Porto 4099-002, Portugal; ⊥ INEB − Instituto de Engenharia Biomédica, Universidade do Porto, Porto 4099-002, Portugal; # ICBAS−Instituto de Ciências Biomédicas Abel Salazar, Universidade do Porto, Porto 4099-002, Portugal; ∇ Department of Biotechnology and Food Engineering, Technion − Israel Institute of Technology, Haifa 32000, Israel; ○ IUCS-CESPU − Instituto Universitário de Ciências da Saúde, Gandra 4585-116, Portugal

**Keywords:** cell-free protein synthesis systems, ATP energy regeneration, phosphoserine, protein expression, synthetic cells, α-synuclein

## Abstract

Energy-supplying molecules are essential for biological processes, particularly for transcription and translation. Cell-free protein synthesis (CFPS) systems are powerful tools for *in vitro* protein production, offering flexibility for applications ranging from high-throughput protein screening to therapeutic protein production. However, energy regeneration in CFPS remains a key challenge, particularly for large-scale or resource-constrained settings. In this study, we introduce phosphoserine (PS) as a simple, cost-effective alternative secondary energy source, capable of partially or fully replacing 3-phosphoglycerate (PGA), the commonly used energy donor in *E. coli* lysate-based CFPS, whose availability is often limited. By supplementing CFPS reactions with PS, we demonstrate significant improvements in protein yield and cost-efficiency, achieving a 2-fold increase in protein production. Importantly, PS enhancement is maintained across lysate batches and protein targets. Furthermore, we offer affordable CFPS compositions that retain protein synthesis, making the system more accessible for resource-limited settings. Additionally, we show that higher PS concentrations, while reducing final protein yield, extend the reaction duration by more than 2-fold. Therefore, the incorporation of PS as an alternative energy donor enables a tunable modality for balancing protein yield, reaction longevity, and cost. Our data support a model in which PS enhances CFPS via the serine biosynthesis pathway by modulating flux between serine production and glycolysis to support ATP regeneration. Lastly, we validate the use of these optimized CFPS compositions within synthetic cells (SCs). This study establishes PS as a promising energy source for *E. coli* lysate-based CFPS systems, paving the way for enhanced and economical protein synthesis platforms tailored to diverse clinical, biotechnological, and industrial needs.

## Graphical abstract

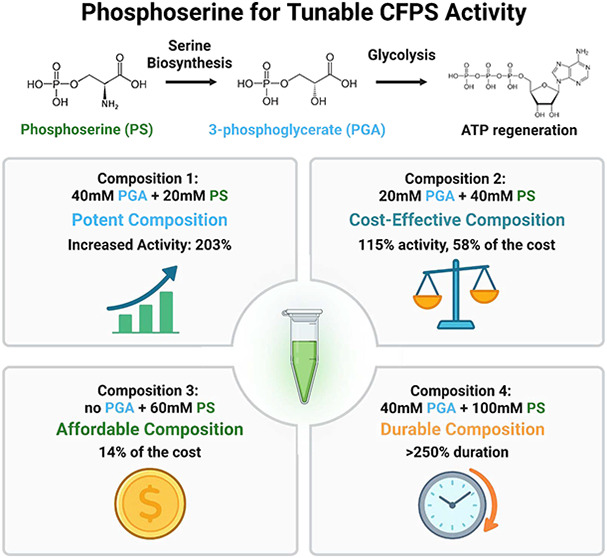

## Introduction

Cell-free protein synthesis (CFPS), also known as *in vitro* transcription-translation (TX-TL), is a versatile lysate-based system for producing RNA and proteins from a DNA template. This system incorporates the essential molecular machinery required for transcription and translation, such as RNA polymerase, ribosomes, and tRNA, provided by a lysate, with externally supplemented nucleotides, amino acids, salts, and energy sources (Figure 1A).^1–5^ While *E. coli* extract is the most widely used lysate,^5^ other sources such as plant, mammalian, and insect cell extracts are also commonly utilized.^1,6–9^

**Figure 1:**
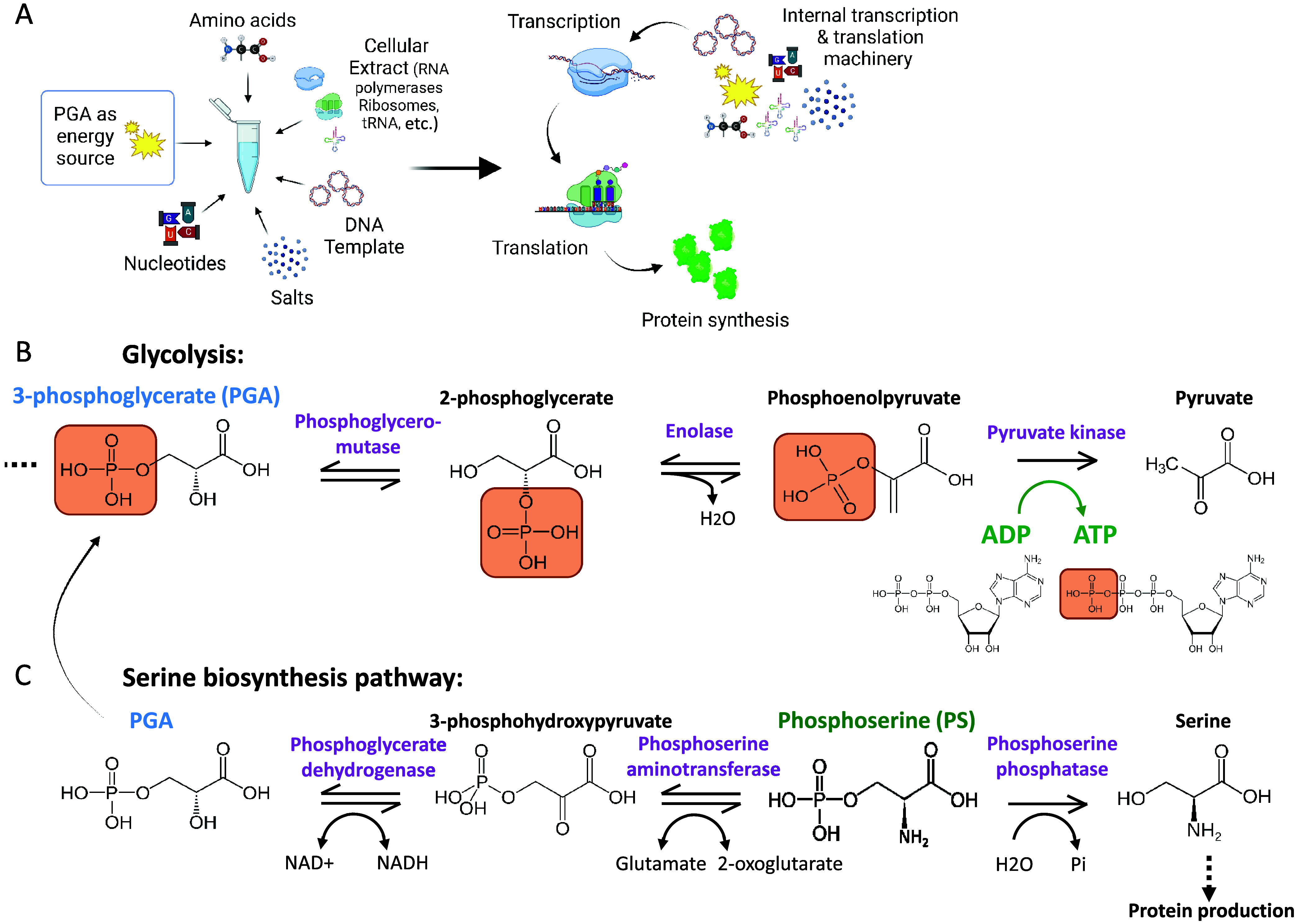
PGA and PS as energy sources for CFPS reactions. (A) A schematic illustration of an *E. coli*-based CFPS system reaction with PGA as energy source. (B) A schematic illustration of PGA as a means of recycling ADP to ATP as part of the glycolysis process. (C) A schematic illustration of the phosphorylated serine biosynthesis pathway.

CFPS systems, unburdened by the need to support cellular viability and growth, allow resources to be fully dedicated to the production of desired proteins.^6^ Originally developed to explore evolutionary processes and the origins of life,^10^ CFPS offers the potential to surpass the limitations of living systems, enabling the production of proteins toxic to cells and functioning in extreme environments such as space. Over the last two decades, CFPS has become a valuable tool in synthetic biology, nanotechnology and biomedical research, demonstrating its versatility in applications ranging from high-throughput protein expression^10^ and screening methods^11,12^ to therapeutic protein production^4,13^ and large-scale antibody synthesis.^14^

Synthetic cells (SCs), which encapsulate CFPS systems within bilayer lipid vesicles, further illustrate the versatility of CFPS systems.^4,15,16^ SCs provide high modularity, the ability to produce both natural and synthetic proteins within cell-like particles, precise control over protein production and release, and communication with natural cells.^16–20^ Their adaptable design, including size, activity, internal composition, and membrane properties, offers significant potential for clinical applications.^4,16,21–24^

Despite these capabilities, the widespread adoption of CFPS and SCs remains constrained by the cost and complexity of energy regeneration. This limitation is particularly pronounced in educational settings and field-based applications, where affordability, simplicity, and robustness are critical. Current ATP regeneration techniques for CFPS systems and SCs, although effective, are complex and depend on multiple enzymes and cofactors, along with structural modifications to the SCs.^25–34^

In CFPS systems, as in living cells, ATP functions as the primary energy currency, playing a crucial role in protein synthesis. The high energy demand of translation, which consumes the equivalent of five ATP molecules per amino acid, highlights the importance of maintaining adequate ATP levels to ensure system efficiency.^5,35^ Researchers have explored various secondary energy sources with high-energy phosphate bonds to sustain the ATP levels required for the transcription and translation processes in CFPS systems.^35,36^ These efforts have leveraged various metabolic pathways, including substrates from glycolysis and substrate-level phosphorylation. Early CFPS systems used energy substrates such as pyruvate,^37,38^ glucose-6-phosphate,^37,39^ and phosphoenol pyruvate.^40,41^ Currently, 3-phosphoglycerate (PGA), a glycolysis metabolite, is widely used secondary energy source due to its high efficiency in ATP recycling, resulting in extended protein production and higher yields^36,37,42–45^ (Figure 1B).

Even with a secondary energy source, the high energy demands of transcription and translation make energy supply a major limiting factor in the efficiency of CFPS systems.^43,44,46,47^ Producing 1 mg/mL of an average-sized protein requires approximately 40 mM of ATP.^35^ Moreover, the high cost of nucleoside triphosphates and phosphate energy donors, key components in CFPS^5,39^ limits the widespread use of CFPS systems (Supplementary Table S1).

**In this work, we present a simple, durable and cost-effective alternative for ATP regeneration in crude** ***E. coli*** **lysate-based cell-free metabolism. We demonstrate that phosphoserine (PS) can efficiently support protein synthesis, partially or fully replacing PGA**. PS is a natural amino acid derived from PGA, a key metabolite in the phosphorylated pathway of serine biosynthesis,^48,49^ making it available and inexpensive (Figure 1C). In this work, we demonstrate that higher concentrations of PS, while resulting in lower final protein production, can prolong the reaction duration by more than 2-fold (Figure 2). Since the *E. coli* lysate already contains all the necessary proteins, PS supports CFPS functionality without the need for additional enzymes. This metabolite can, when combined with PGA, double the cost-effectiveness of a CFPS reaction by enhancing protein yield and reducing overall costs. We introduce an alternative composition that can increase protein production 2-fold. Importantly, PS enhances CFPS performance across independently prepared lysate batches and multiple protein targets, with varied efficacy. We also describe a lower cost composition (15% of the original reaction) maintaining 20% of the activity. This reveals a tunable mechanism for balancing protein yield, reaction longevity and cost, allowing for tailored optimization to meet specific application requirements. Finally, we validate the effectiveness of this novel CFPS composition within SCs. These advancements represent a major step toward making CFPS systems more accessible and cost-effective, opening up broader applications in research and industry.

**Figure 2:**
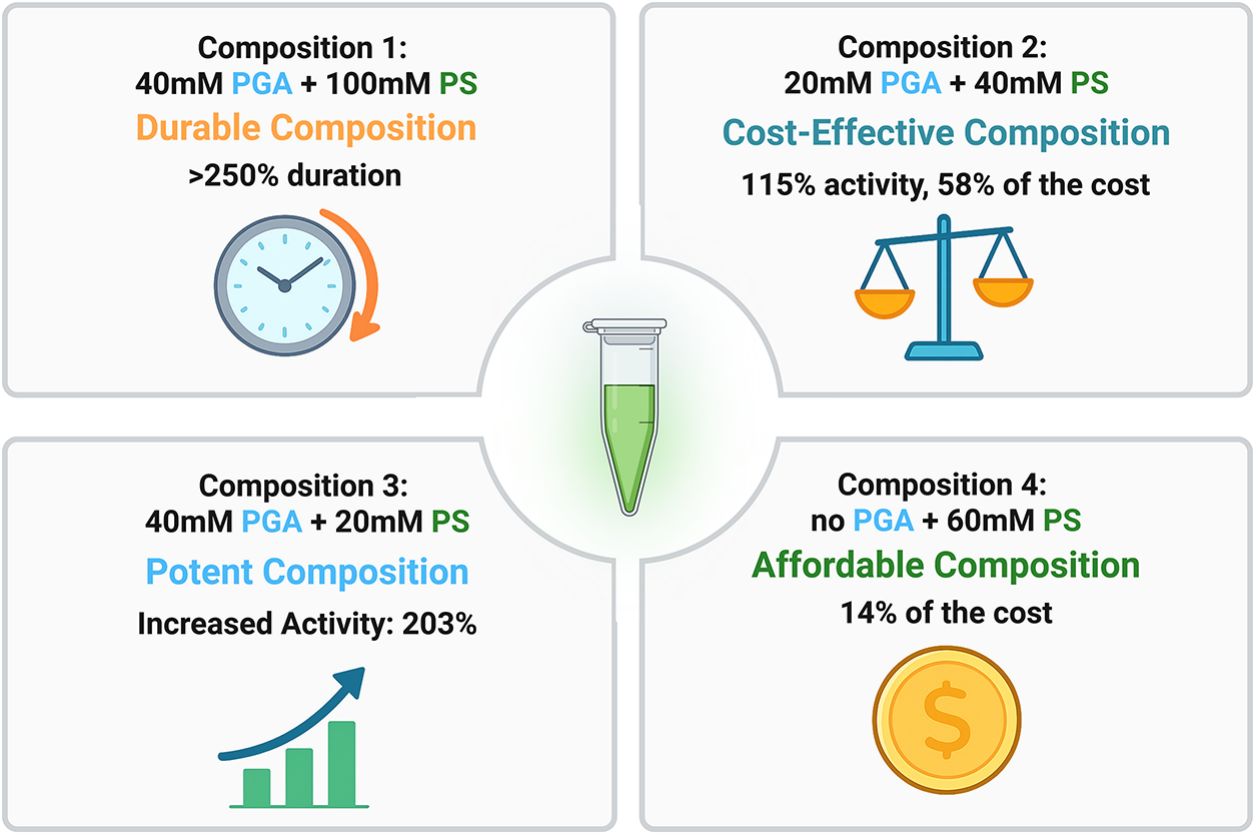
Comparative performance of PS and PGA energy-regeneration compositions in *E. coli* lysate-based CFPS.

## Results and Discussion

### Phosphoserine (PS) and 3-Phosphoglycerate (PGA) as Secondary Energy Sources

In CFPS reactions, two energy sources are used: ATP, which is directly consumed during transcription and translation, and a secondary energy source, which supports ATP regeneration from ADP. Using an *E. coli*-based CFPS system with PGA, a commonly used secondary energy source, we optimized ATP and PGA concentrations for protein expression, as both are critical and costly components (Supplementary Table S1). Superfolder green fluorescent protein (sfGFP) production increases with ATP and PGA concentrations, peaking at 2.4 mM ATP and 70 mM PGA (Supplementary Figure S1). In the absence of PGA, protein expression ceases, underscoring its essential role in the reaction. However, for both components, higher concentrations gradually reduce production, eventually stopping it altogether.

**PS is a natural amino acid derived from PGA (Figure 1C). Given the essential role of PGA in CFPS reactions, we explored the potential of PS as an alternative secondary energy source**. PS molecular distinction from PGA was confirmed using a ninhydrin assay, thin-layer chromatography (TLC), and nuclear magnetic resonance (^1^H NMR)^50^ (Supplementary Figures S2–S4).

Increasing concentrations of PS were added to sfGFP-producing CFPS reactions as the sole secondary energy source. PS displayed a similar trend to ATP and PGA, although with lower sfGFP production (Figure 3A­(i)). The system’s activity improved with increasing PS concentrations, peaking at 80 mM, beyond which higher concentrations hindered protein production. **These results demonstrate that PS can fully replace PGA as a secondary energy source in CFPS, although with reduced protein yield.**

**Figure 3:**
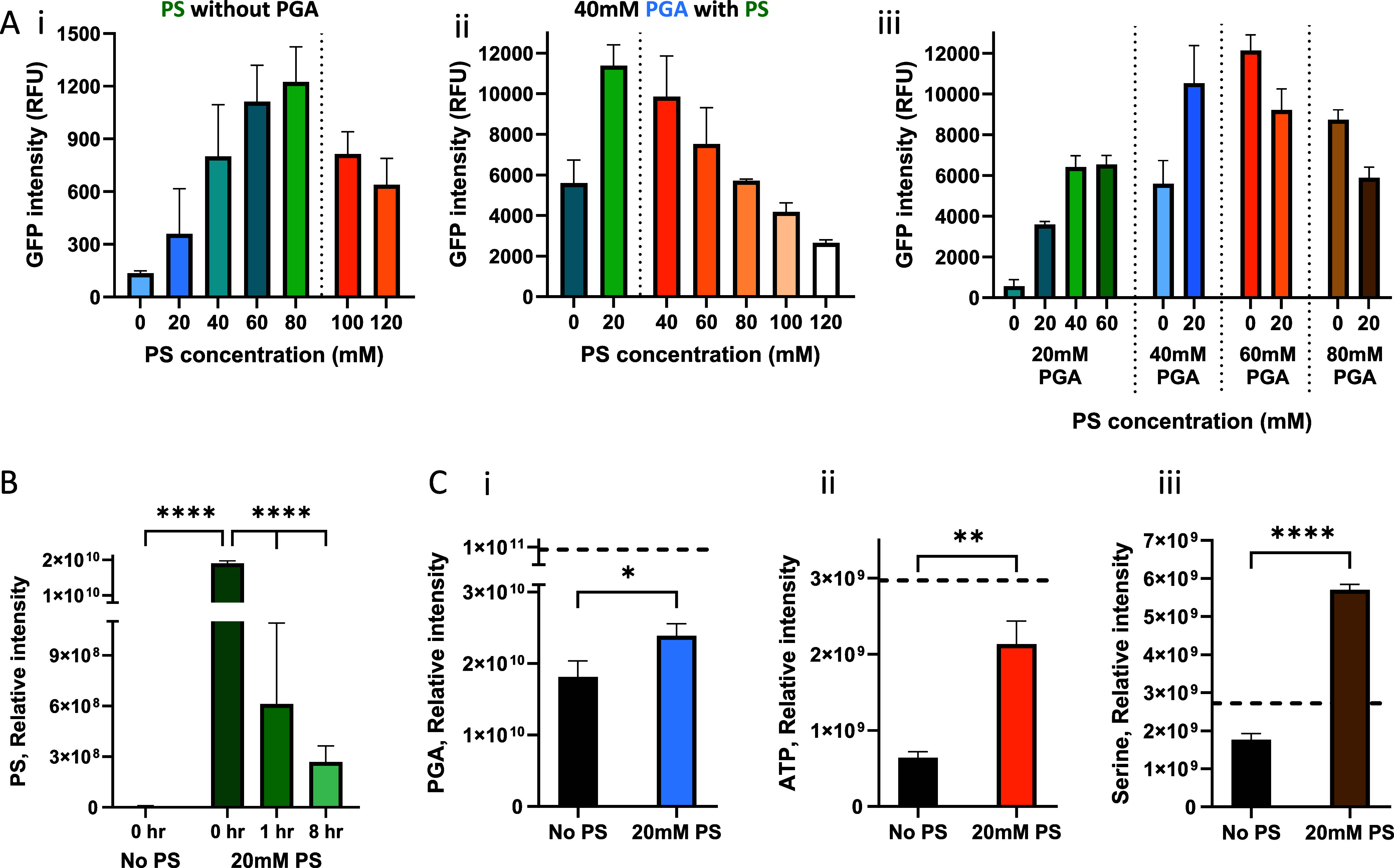
Phosphoserine (PS) as a secondary energy source for CFPS reactions. (A) sfGFP production over 8 h in an *E. coli*-based CFPS reaction with different concentrations of PS: (i) without PGA, (ii) with 40 mM PGA and (iii) with varying concentrations of PGA. Data are expressed as mean ± s.e.m (*n* = 4–8 independent samples). (B) LC-MS analysis of relative PS levels in CFPS reactions without PS and with 20 mM PS at different time points (0, 1, and 8 h). Data are expressed as mean ± s.e.m. Two-way ANOVA with adjusted *P* value in Tukey’s multiple comparisons tests, ****P = <0.0001. (*n* = 3 independent samples). (C) LC-MS analysis of relative levels of (i) PGA, (ii) ATP, and (iii) serine in CFPS reactions with and without 20 mM PS, measured shortly after mixing. Dashed lines indicate metabolite levels in reactions lacking *E. coli* lysate, corresponding to the initial concentrations added. Data are presented as mean ± s.e.m. Unpaired two-tailed *t* test *P* value, *P = 0.0332, **P = 0.0021, ****P = <0.0001 (*n* = 3 independent samples).

### PS and PGA Combinations for Efficient Protein Production

We evaluated the efficiency of combining PS and PGA in CFPS reactions. We found that specific PS and PGA combinations can significantly improve protein production beyond levels achieved with PGA alone (Figure 3A). Adding 20 mM PS to a reaction containing 40 mM PGA doubled sfGFP production, achieving (11.4 ± 0.3) × 10^3^ relative fluorescent units (RFU) compared to (5.6 ± 0.3) × 10^3^ RFU with 40 mM PGA alone (Figure 3A­(ii)), corresponding to $\left(0.61\pm 0.03\right)\frac{\mathrm{mg}}{\mathrm{mL}}\;\mathrm{and}\;\left(1.24\pm 0.03\right)\frac{\mathrm{mg}}{\mathrm{mL}}$ , respectively (Supplementary Figure S5). However, higher PS concentrations inhibited the reaction, consistent with observations for each metabolite individually (Figure 3A­(ii), Supplementary Figure S1). A similar trend was observed for the production of monomeric red fluorescent protein 1 (mRFP1) (Supplementary Figure S6).

The addition of PS to reactions containing PGA concentrations higher than 40 mM did not enhance protein production and instead replicated the inhibitory effects (e.g., adding 20 mM PS to 60 mM PGA reduced GFP intensity from (12.1 ± 0.2) × 10^3^ RFU to (9.2 ± 0.4) × 10^3^) (Figure 3A­(iii)).

While the combination of 40 mM PGA and 20 mM PS resulted in the highest activity, two other PGA and PS combinations also improved protein expression relative to 40 mM PGA alone: reactions containing 20 mM PGA and either 40 mM or 60 mM PS produced GFP intensities of (6.4 ± 0.3)*10^3^ RFU and (6.5 ± 0.2)*10^3^ RFU, respectively, representing a 15% improvement over the original composition while using less PGA (Figure 3A­(iii)).

### Metabolic Role of PS in *E. coli*-based CFPS Reactions

As a natural amino acid derived from PGA, PS is a key intermediate in the phosphorylated serine biosynthesis pathway (Figure 1C).^49,51–56^ To directly assess the role of PS in CFPS metabolism, we quantified key pathway intermediates using LC-MS analysis (Figure 3B,3C, Supplementary Figure S7). PS was not detected in reactions lacking PS supplementation, confirming that it is not present in the baseline system (Figure 3B). In PS-supplemented reactions, PS levels decreased over time, consistent with our hypothesis that it is consumed during the reaction. In addition, levels of PGA and ATP were significantly higher in reactions containing PS compared to reactions without PS (Figure 3C­(i–ii)). These results suggest that PS increases the availability of intermediates feeding into glycolysis, consistent with PS conversion to PGA through the serine biosynthesis pathway and subsequent contribution to ATP regeneration.

Serine levels were also significantly elevated in PS supplemented reactions, exceeding the initial concentration added to the reaction (Figure 3C­(iii)), supporting downstream conversion of PS through the serine biosynthesis pathway.

To further examine the mechanism driving this effect, we conducted the following experiments: (A) CFPS reactions containing 40 mM PGA with or without 20 mM PS, supplemented with increasing concentrations of glutamate. Since glutamate is a product of PS conversion to 3-phosphohydroxypyruvate by phosphoserine aminotransferase, this experiment aimed to test whether it hinders protein synthesis in the presence of PS, potentially by directing enzyme activity downstream in the serine biosynthesis pathway (Figure 1C). Indeed, in the absence of PS, glutamate had little effect on protein production, whereas in its presence, it caused a noticeable slower rate of activity (Supplementary Figure S8). (B) CFPS reactions containing 40 mM PGA, supplemented with increasing serine concentrations, to determine whether serine alone, like PS, could enhance protein synthesis. Indeed, serine increased sfGFP production (Supplementary Figure S9­(i)), supporting the hypothesis that the enhancement by PS stems from its involvement in serine biosynthesis. (C) CFPS reactions without serine (typically included in the amino acid mixture of the CFPS solution) with increasing PS concentrations, to assess whether PS could compensate for its absence. As expected, PS fully restored protein synthesis even without serine (Supplementary Figure S9­(ii)), reaching levels comparable to reactions containing both serine and PS (Figure 3A­(ii)).

**Together, these results strongly suggest that PS enhances CFPS through the serine biosynthesis pathway, thereby supporting its role as an alternative energy source**. Two complementary mechanisms may account for the observed increase in ATP production. First, conversion of PS to PGA. Due to the near-equilibrium nature of 3-phosphoglycerate dehydrogenase and phosphoserine aminotransferase enzymes, PS can be converted to PGA.^54,57–60^ The resulting PGA can then enter glycolysis and contribute to ATP generation. The results of the first and third experiments support this mechanism. Second, PS may reduce the diversion of PGA into serine biosynthesis, preserving more PGA for glycolytic flux and ATP production. This interpretation is consistent with the second experiment and with literature indicating that phosphoserine phosphatase catalyzes an irreversible step, while upstream reactions operate near equilibrium.^54,57–62^

The combination of these effects enables PS to modulate the metabolic flux between serine biosynthesis and glycolysis in CFPS, thereby enhancing ATP production and overall system performance. Moreover, since the *E. coli* lysate used already contains the necessary enzymes for this pathway, PS supports CFPS functionality without requiring the addition of external enzymes. **This enables more efficient utilization of the existing protein machinery, maximizing the system’s metabolic potential.**

### PS for Sustaining Long-Lasting CFPS Activity

**During CFPS protein production with PGA as the sole secondary energy source, without PS added, protein expression plateaus at approximately 200 min, regardless of the initial ATP or PGA concentrations (** Supplementary Figure S10 **)**. To test whether enzymatic inactivation caused the observed plateau, the *E. coli* lysate (the active reaction components) was preincubated under the experimental conditions for varying durations. The preincubated lysate was then added to a complete CFPS reaction mixture. Enzymatic activity was maintained and slightly enhanced, demonstrating that the observed plateau does not result from gradual loss of enzymatic activity (Supplementary Figure S11).

An alternative explanation is that CFPS activity plateaus due to a decline in the available energy within the system. To evaluate this possibility, we measured ATP concentrations at different time points during the CFPS reaction. Consistent with this hypothesis, ATP concentrations decreased as the reaction progressed and sfGFP was produced (Figure 4A-black lines).

**Figure 4:**
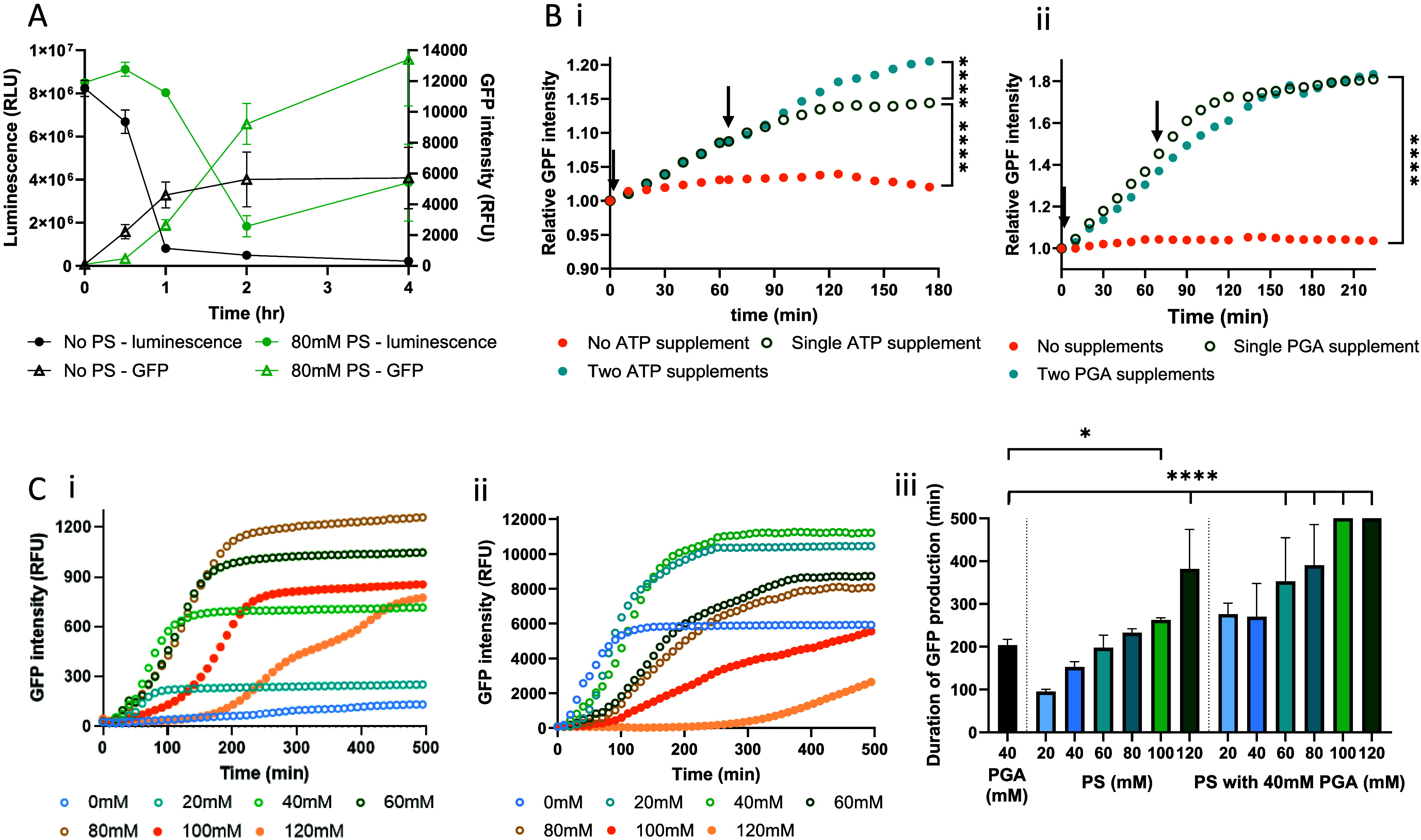
PS as a durable energy source. (A) ATP depletion and sfGFP production over time in CFPS reactions containing 40 mM PGA with or without 80 mM PS. ATP concentrations (luminescence, left axis) and sfGFP expression (fluorescence, right axis) were measured at various time points (0, 0.5, 1, 2 and 4 h). Data are expressed as mean ± s.e.m (*n* = 3 independent samples). (B) The effect of energy source supplementation on CFPS activity, after sfGFP production plateaued. The additions of the energy sources are marked by arrows. The initial concentrations in the reaction were 1.2 mM ATP and 40 mM PGA. (i) 1.2 mM ATP supplementations. (ii) 40 mM PGA supplementations. All values are normalized to the amount of sfGFP in the solution at the time of the first addition. Time 0 indicates the first supplementation to the test samples after GPF production plateaued. Two-way ANOVA with adjusted P value in Tukey’s multiple comparisons tests, ****P = <0.0001 (*n* = 5–6 independent samples). (C) sfGFP production over time in a CFPS reaction with different initial concentrations of PS, with (i) no PGA, and (ii) 40 mM PGA (*n* = 4–7 independent samples). (iii) Effect of PS and PGA concentrations on the duration of sfGFP production in CFPS reactions. Two-way ANOVA with adjusted P value in Dunnett’s multiple comparisons tests, *P* = >0.1234, *P = 0.0332, ****P = <0.0001 (*n* = 3–7 independent samples).

To address this limitation, we explored energy source supplementation during CFPS reaction, after protein production had plateaued. A single ATP supplementation increased sfGFP production by (14.4 ± 1.6)% (Figure 4B­(i)). A second ATP supplementation further enhanced protein output, increasing GFP intensity by an additional (6.1 ± 2.6)%, reaching to total of (20.5 ± 2.1)% compared to the unsupplemented reaction. In contrast, PGA supplementation resulted in significantly larger increase in sfGFP production, with an (81.6 ± 23.3)% improvement over the unsupplemented reaction (Figure 4B­(ii)). However, a second PGA addition barely yielded further improvement (increasing GFP intensity by an additional (2.8 ± 29.0)%, reaching to total of (84.4 ± 17.3)% improvement over the unsupplemented reaction). Supplementation of other reaction components did not replicate these gains (Supplementary Figure S12). These findings indicate that the protein production plateau primarily results from ATP depletion, and that the ability of PGA supplementation to prolong synthesis is limited to a single addition.

**Interestingly, at Higher Concentrations, PS Extended the Duration of Protein Production without the Need for Supplementation.**

PS concentrations of 80 mM or higher, in the absence of PGA, prolonged the active period beyond 200 min (Figure 4C­(i)). Specifically, the activity of CFPS reactions containing 80 mM PS was extended by 30 min, while 100 mM and 120 mM PS sustained protein synthesis for 260 and 380 min, respectively. Notably, increasing the energy source concentration reduced the final GFP intensity, indicating a trade-off between activity duration and protein yield.

When we examined protein production in reactions containing **combinations of PS and PGA over time**, we observed the same extended activity as in reactions with PS alone, but with higher yields (Figure 4 **C­(ii)** and Supplementary Figure S13). Reactions containing 20 mM PGA and 60 mM PS extended activity to 300 min, while reactions with 40 mM PGA and 60 mM and 80 mM PS maintained activity for 350 and 390 min, respectively (Figure 4C­(iii)). **For reactions with 40 mM PGA and 100 mM or 120 mM PS, protein synthesis persisted beyond 500 min**.

The extended CFPS activity may be explained by slower ATP regeneration, which prolongs energy availability over time. While PGA directly fuels glycolysis, PS must first convert to PGA via the serine biosynthesis pathway. When we measured ATP concentrations in CFPS reactions containing 40 mM PGA, with or without 80 mM PS, ATP depletion in the presence of PS was significantly slower compared to reactions without PS (Figure 4A and Supplementary Figure S14).

A potential key factor influencing ATP regeneration from PS may be the relative abundance of enzymes in the glycolytic and serine biosynthesis pathways. We compared the relative abundance of enzymes from the serine biosynthesis pathway and glycolysis (Supplementary Figure S15). In contrast to the relatively low abundance of serine biosynthesis enzymes, glycolytic enzymes were present at substantially higher levels. This enzymatic profile may result in a gradual and sustained release of PGA into glycolysis, extending ATP regeneration compared to direct PGA supplementation. Consequently, this slower, sustained ATP regeneration is consistent with the prolonged CFPS activity observed at higher PS concentrations. When PS is combined with PGA, the system benefits from both immediate ATP recycling via PGA and extended activity through slower PS-to-PGA conversion, leading to higher overall protein yields over time.

**Extending the Active Period of CFPS Reactions Offers Numerous Potential Benefits**. In therapeutic applications, for instance, prolonged activity could support sustained protein production for replacing malfunctioning cells or synthesizing therapeutic proteins in situ. Similarly, this extension could enhance the accuracy of studies exploring evolutionary processes, enzymatic pathways, diagnostics, or biosensor applications. Additionally, prolonging CFPS activity enables more efficient utilization of reagents, potentially reducing the cost per unit of protein produced.

**These findings demonstrate that specific combinations of PS and PGA significantly extend the duration of CFPS activity.**

### The Cost-effectiveness of PS as An Energy Source

The price of available PGA rose significantly between 2023 and 2024 (Figure 5A and Supplementary Table S1 **)**. In 2023, PGA accounted for 68.5% of the total cost of a CFPS reaction. However, in 2024, the price increase in PGA caused the overall cost of a CFPS reaction to rise by 276%, with PGA comprising 88.6% of the total cost. **In contrast, PS is currently priced at only 2.6% of the cost of PGA per gram, making it a much more affordable secondary energy source.**

**Figure 5:**
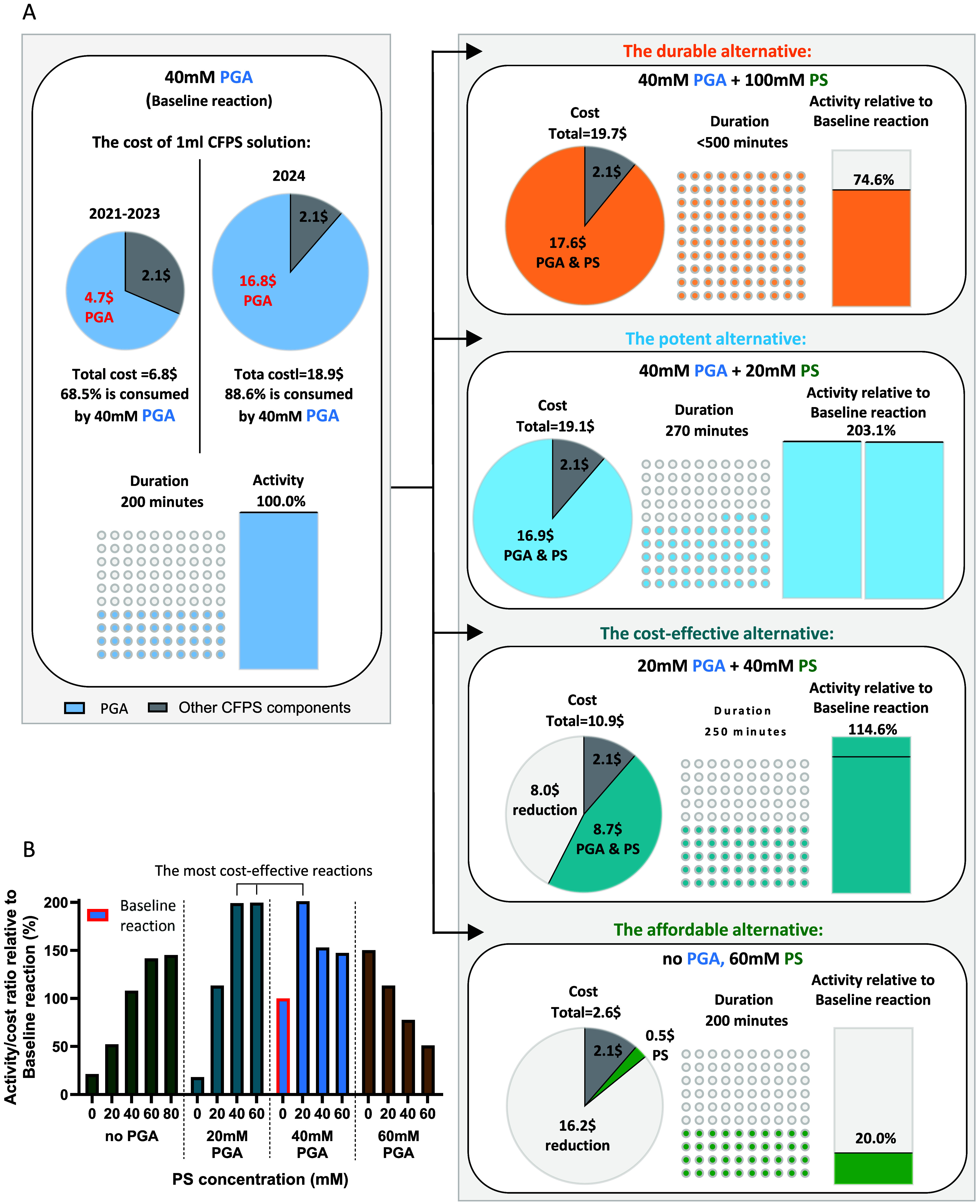
PS as a cost-effective secondary energy source for CFPS reactions. (A) An illustration of PS as an energy source in the durable, affordable, cost-effective and potent CFPS compositions. (B) The activity-to-cost ratio of CFPS reactions with varying concentrations of PGA and PS, normalized to the baseline reaction (40 mM PGA).

To quantify the cost-effectiveness of CFPS reactions, we defined **efficiency** as the ratio of GFP intensity to reaction cost $\left(e\mathrm{fficiency}=\frac{\mathrm{activity}}{\mathrm{cost}}\right)$ . Figure 5B and Supplementary Table S2 compare the efficiency of CFPS reactions with varying concentrations of PGA and PS. The baseline relative efficiency, set at 100%, corresponds to a reaction with 40 mM PGA serves as the sole secondary energy source.

When PS was used as the sole secondary energy source, efficiency increased with rising PS concentrations, peaking at 145.2% with 80 mM PS. The combination of PS with 20 mM PGA led to even greater efficiency gains. The combination of 20 mM PGA and 40 mM PS showed the highest efficiency at the lowest cost among the top-performing alternatives. This condition uses the lowest concentrations of both PGA and PS, making it **the most cost-effective option** (Figure 5A and Table 1).

**Table 1:**
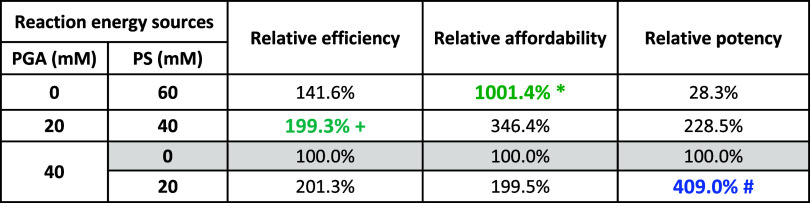
Efficiency, Affordability, and Potency of CFPS reactions using PS as an energy source^a^,^b^,^c^,^d^

a Gray background—baseline reaction.

b **Cyan**- +—the alternative with the highest efficiency at the lowest cost, $e\mathrm{fficiency}=\frac{\mathrm{activity}}{\mathrm{cost}}$ .

c **Green**- *—the alternative with the lowest cost relative to its efficiency, $\mathrm{affordability}=\frac{\mathrm{efficiency}}{\mathrm{cost}}$ .

d **Blue**- #—the alternative with the highest activity relative to its efficiency, potency = efficiency × activity.

In certain scenarios, affordability may take precedence over efficiency. For example, CFPS reactions are often used in educational settings due to their simple setup and rapid execution, requiring detectable outputs at the lowest possible cost to ensure accessibility.^63–66^ Similarly, high-throughput protein screening methods can benefit from such systems.^11,12^ To minimize the cost of CFPS reactions while maintaining efficiency, we defined **affordability** as the ratio of efficiency to reaction cost $\left(a\mathrm{ffordability}=\frac{e\mathrm{fficiency}}{\mathrm{cost}}=\frac{activity}{\cos t^{2}}\right)$ (Supplementary Table S3). We set the baseline relative affordability at 100%, corresponding to a reaction with 40 mM PGA. A reaction with 60 mM PGA maintained comparable affordability to the baseline (104.1%). Adding PS to this reaction decreased affordability down to 34.8% for 60 mM PS. Interestingly, the addition of PS to lower PGA concentrations significantly improved affordability. For example, a reaction with 20 mM PGA and no PS showed affordability of 32.6%, but the addition of 40 mM PS increased affordability to 346.4%. Remarkably, in reactions without PGA, affordability increased dramatically, with 20 mM, 40 mM, and 60 mM PS yielding affordability levels of 424.4%, 817.7%, and 1001.4%, respectively. These results suggest that the reaction with 60 mM PS offers the lowest cost relative to its efficiency, making it **the most affordable option (** Figure 5A and Table 1 **)**.

In contrast, applications such as therapeutics and large-scale protein synthesis may prioritize maximizing protein yield over cost. To maximize the activity of CFPS reactions relative to their efficiency, we defined **potency** as the multiplication of reaction activity and efficiency $\left(\mathrm{potency}=\mathrm{efficiency}\times \mathrm{activity}=\frac{\mathrm{activit}y^{2}}{\cos t}\right)$ . Reactions without PGA exhibited very low potency, reaching only 31.7% at 80 mM PS. However, the addition of PS to PGA reactions improved potency significantly (Supplementary Table S3). The combination of 40 mM PGA with 20 mM PS was **the most potent alternative**, offering the highest protein output relative to its efficiency, with a potency of 409.0% **(** Figure 5A and Table 1 **)**.

**In conclusion, PS provides a flexible approach to optimizing affordability and protein yield in** ***E. coli*** **lysate-based CFPS reactions.**

### Optimized CFPS Compositions for Tunable Protein Expression in SCs

Encapsulating cell-free reactions enables the integration of protein production with membrane functionality, offering potential in biomedical and therapeutic applications, as well as facilitating communication with biological cells. We examine the incorporation of our optimized CFPS compositions, utilizing PS as an energy source, within SCs (Figure 6A). **We compared sfGFP production in SCs under three conditions: a baseline reaction (40 mM PGA), the most potent combination (40 mM PGA and 20 mM PS), and the most affordable composition (60 mM PS)**, following 2 h of protein synthesis at 37 °C. As expected, the potent combination increased sfGFP production inside SCs, from (2.1 ± 0.6) × 10^5^ RFU in the baseline reaction to (3.1 ± 0.6) × 10^5^ RFU (Figure 6B). Although the affordable 60 mM PS reaction yielded less activity, with (0.67 ± 0.04) × 10^5^ RFU, the protein production was detectable after 2 h. When we examined protein production within the SCs over time, we observed an extended period of activity consistent with that seen in bulk CFPS reactions (Figure 6C). Both the baseline and potent formulations remained active for approximately 190 min, whereas the affordable formulation remained active for an additional 60 min, up to 250 min.

**Figure 6:**
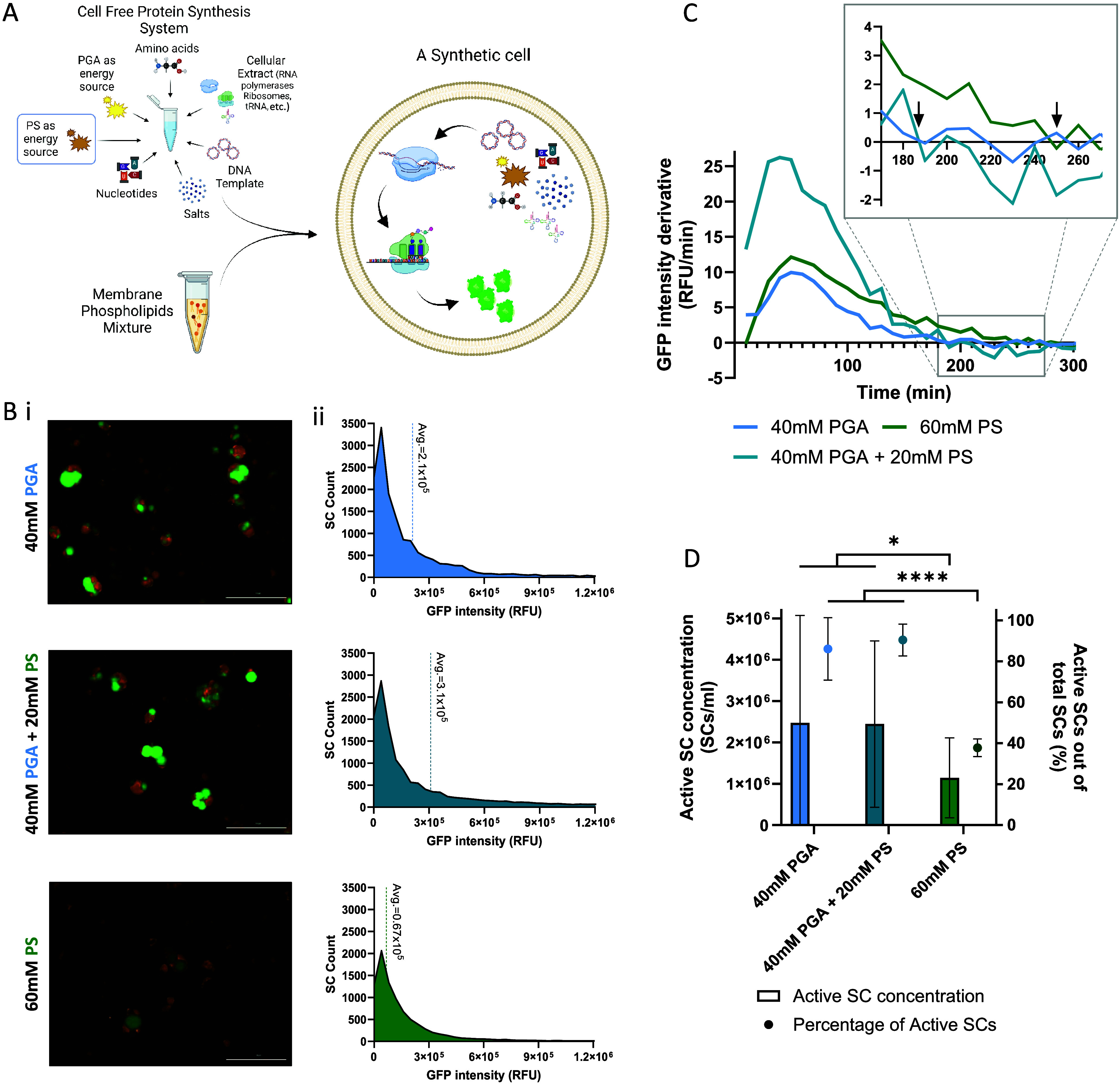
PS as an energy source for synthetic cells (SCs). (A) Schematic illustration of protein-producing SCs: the CFPS system solution is encapsulated within a synthetic phospholipids membrane composed of POPC and cholesterol in 1:2 molar ratio. (B) sfGFP production in different SC populations with varying PGA and PS concentrations after 2 h of protein expression at 37 °C. (i) Representative fluorescence images of sfGFP-producing SCs; *produced sfGFP (green), Rhodamine–labeled membrane (orange).* The images were captured using a fluorescent microscope with RFP and GFP filters, merged. (ii) GFP intensity distribution of active SC populations (*n* = 5 independent samples). (C) Derivative of sfGFP production over time in protein-producing SCs with different initial PS and PGA concentrations. Arrows indicate the time points at which the GFP intensity derivatives reached zero (*n* = 4 independent samples). (D) Concentration and percentage of active SC populations produced with varying PGA and PS concentrations. Data are expressed as mean ± s.e.m. Two-way ANOVA with adjusted P value in Tukey’s multiple comparisons test, *P* = >0.1234, *P = 0.0332, ****P = <0.0001 (*n* = 5 independent samples).

Total SC concentrations were similar across all reactions, (14.4 ± 3.7) × 10^6^ SCs/mL for the baseline reaction, (17.5 ± 3.1) × 10^6^ SCs/mL for the potent combination, and (18.5 ± 3.9) × 10^6^ SCs/mL for the affordable alternative. Active SC concentrations and percentages were also similar between the baseline reaction and the potent reaction, with (2.5 ± 0.9) × 10^6^ SCs/mL and 86 ± 5% for the baseline reaction, and (2.4 ± 0.6) × 10^6^ SCs/mL and 90 ± 2% for the potent combination (Figure 6D). The affordable reaction showed lower active SC concentration and percentage, at (1.1 ± 0.3) × 10^6^ SCs/mL and 38 ± 1%, likely due to its reduced activity. **These findings highlight the adaptability of CFPS compositions in SCs: the potent combination enhances protein output, making it suitable for high-yield applications like therapeutics, while the affordable alternative offers a cost-effective solution for settings where affordability is key, such as education**.

### Consistency of the PS Effect

**Establishing the practical utility of PS requires demonstrating consistent enhancement of CFPS protein production across experimental conditions**. Given the variability in lysate composition between preparations, we examined whether the effect of PS is consistent across independently prepared lysate batches and different protein products (Figure 7).

**Figure 7:**
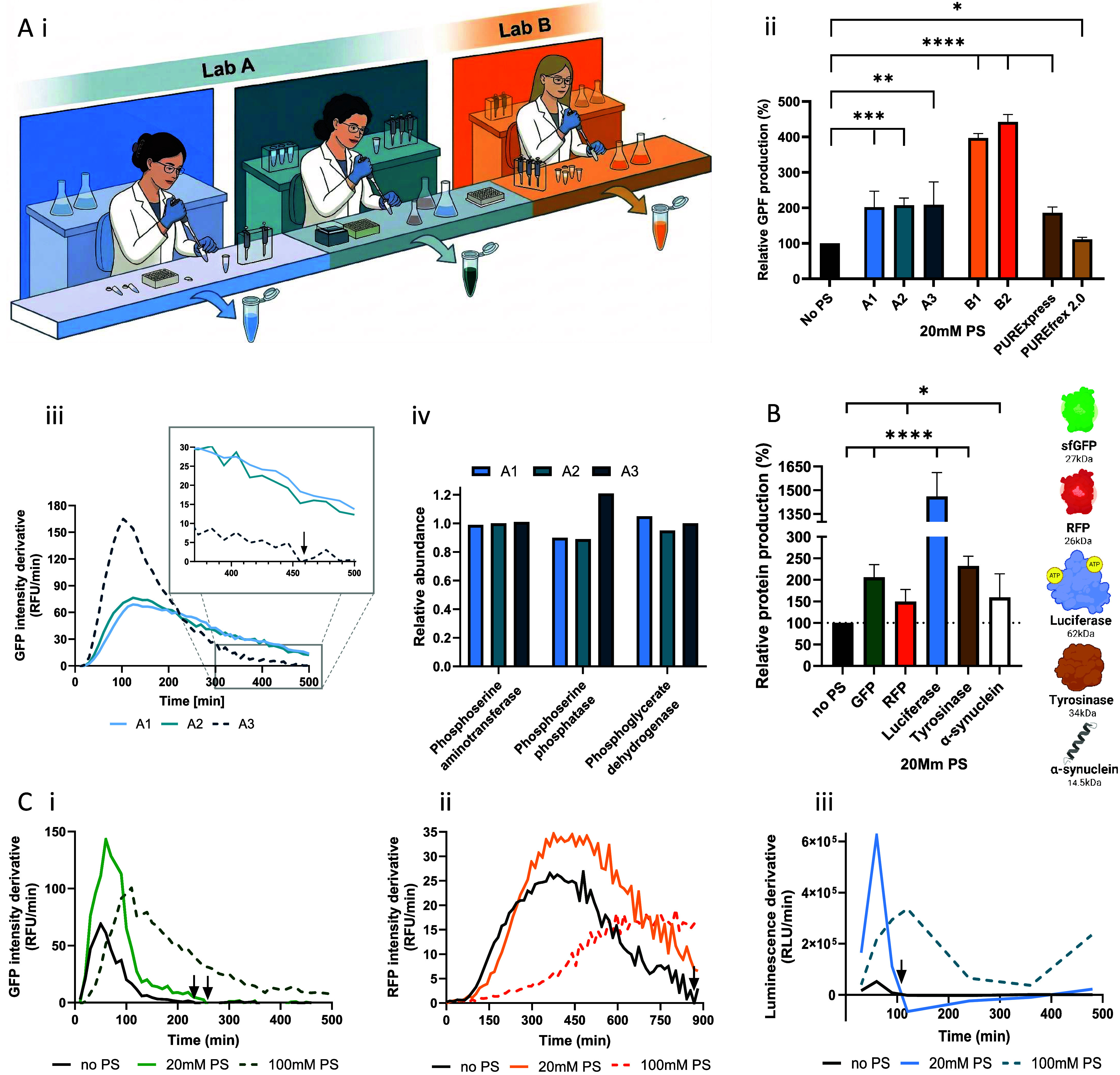
Consistency of the PS effect. (A) sfGFP production in CFPS reactions using independent lysate batches. (i) Schematic illustration of independent lysate preparation in two laboratories. (ii) Relative sfGFP production in CFPS reactions using independently prepared *E. coli* lysates from two independent laboratories (Lab A, *n* = 3; Lab B, *n* = 2) and in two commercial PURE systems, following supplementation with 20 mM PS. Production is normalized to reactions without PS. Data are expressed as mean ± s.e.m. Unpaired two-tailed *t* test *P* value, *P = 0.0332, **P = 0.0021, ***P = 0.0002, ****P = <0.0001 (*n* = 3–6 independent samples). (iii) Derivative of sfGFP production over time in CFPS reactions using independent lysate preparations with 100 mM PS. The arrow indicates the time point at which the production rate reached zero (*n* = 6 independent samples). (iv) Relative abundance of serine biosynthesis enzymes across independent lysate preparations. Values are normalized to the mean abundance for each protein across lysates. (B) Relative protein production in CFPS reactions expressing different target proteins in the presence of 20 mM PS. Production is normalized to reactions without PS. Data are expressed as mean ± s.e.m. Unpaired two tailed *t* test *P* value, *P = 0.0332, ****P = <0.0001 (*n* = 3–6 independent samples). (C) Protein production rates in CFPS reactions supplemented with 0, 20, or 100 mM PS. Production rates were calculated as the derivative of fluorescence or luminescence intensity over time. Arrows indicate the time point at which the production rate reached zero. (i) sfGFP, (ii) RFP, (iii) Luciferase. (*n* = 3–6 independent samples).

To evaluate lysate-to-lysate variability, we tested the addition of 20 mM PS across independent lysate preparations (Figure 7A­(i)–(ii)). CFPS reactions expressing sfGFP were performed using three *E. coli* lysates independently prepared in our laboratory by different researchers on different days, and two additional lysates prepared in an independent collaborating laboratory. In all lysates tested, supplementation with 20 mM PS significantly increased sfGFP production relative to reactions lacking PS. Lysates prepared in our laboratory showed improvements of (202.0 ± 1.7)%, (207.1 ± 4.2)%, and (208.5 ± 5.9)%, while those prepared in the collaborating laboratory showed larger increases of (396.9 ± 2.6)% and (442.3 ± 0.7)%. Although the magnitude of improvement varied between lysates, all preparations exhibited a clear increase in sfGFP production.

To further evaluate the applicability of PS beyond crude *E. coli* lysate-based CFPS systems, we tested its effect in two commercially available PURE systems, PURExpress (New England Biolabs) and PUREfrex 2.0 (GeneFrontier). Based on the minimal metabolic composition of PURE systems, we expected PS supplementation to have little or no effect on sfGFP expression. Surprisingly, supplementation with 20 mM PS significantly increased protein expression in both systems (by 83.5% in the PURExpress system and by 11.0% in PUREfrex 2.0, Figure 7A­(ii)). These findings suggest that the beneficial effect of PS may not be exclusively dependent on metabolic ATP regeneration and may involve additional mechanisms that remain to be investigated.

We next examined the effect of 100 mM PS on reaction dynamics across the three lysates prepared in our lab (Figure 7A­(iii)). Two of the three lysates prepared in our laboratory displayed sustained sfGFP production throughout the measurement period, whereas one lysate exhibited a plateau at approximately 460 min. Notably, this lysate also showed higher overall production using the 100 mM PS compared to 20 mM PS ((462.8 ± 2.2)% versus (208.5 ± 5.9)% relative to baseline, respectively). This variability likely reflects differences in lysate composition, which can influence metabolic flux through pathways involved in ATP regeneration and protein synthesis. The utilization of PS may depend on the relative abundance of enzymes in the serine biosynthesis pathway. Indeed, while the abundance of Phosphoserine aminotransferase and Phosphoglycerate dehydrogenase were similar between the examined lysates, the lysate showed higher activity with 100 mM PS exhibited 30% higher Phosphoserine phosphatase (Figure 7 **A­(iv)**). **Consequently, while higher PS concentrations consistently prolong CFPS activity, the optimal PS concentration may vary between lysate preparations.**

To determine whether the beneficial effect of PS extends beyond a single reporter protein, we tested CFPS reactions expressing five proteins: sfGFP, RFP, luciferase, tyrosinase and α-synuclein (Figure 7B). Supplementation with 20 mM PS significantly increased protein production for all targets, with improvements of (206.0 ± 28.4)%, (149.6 ± 26.8)%, (1460.8 ± 137.4)%, (232.2 ± 20.5)% and (159.1 ± 39.2)% for sfGFP, RFP, luciferase, tyrosinase and α-synuclein, respectively (Figure 7B­(i)). While sfGFP, RFP, tyrosinase, and α-synuclein showed increases in protein production (approximately 1.5–2.5-fold), luciferase exhibited a markedly higher increase (>10-fold). This difference may reflect increased ATP availability, which can enhance both luciferase expression and enzymatic activity.

To further examine the effect of PS on reaction dynamics across different proteins, we monitored production rates over time for sfGFP, RFP, and luciferase (Figure 7C). In all cases, 100 mM PS prolonged protein production, maintaining activity well beyond reactions containing no PS or 20 mM PS.

**Together, these results demonstrate that PS supplementation broadly enhances and prolongs** ***E. coli*** **-based CFPS performance. At the same time, the magnitude of the effect depends on both lysate composition and the expressed protein. These findings suggest that optimal PS concentrations should be determined for each lysate preparation and target protein**.

## Conclusions

The high energy demands of transcription and translation in CFPS reactions underscore the importance of sustaining adequate energy levels to maintain system efficiency. Here, we show that incorporating PS, a natural amino acid derived from PGA and a key metabolite in the serine biosynthesis pathway, as a secondary energy source in *E. coli* lysate-based CFPS enhances flexibility and cost-effectiveness, either partially or fully replacing PGA. PS supplementation enables tunable control over CFPS performance, as higher PS concentrations significantly extend reaction duration while lower concentrations support higher production rates. Importantly, the beneficial effects of PS are reproducible across independently prepared lysate batches and extend across multiple protein targets, highlighting the generality of this approach. At the same time, the observed variability in response between lysate preparations and protein targets underscores the importance of optimizing PS concentration for each specific system.

This combination of tunability and generality enabled by PS broadens the practical adoption of *E. coli*-based CFPS in resource-limited settings, including educational platforms and low-resource laboratories, where affordability and simplicity are critical. Moreover, the ability to control protein yield and reaction longevity creates new opportunities for the design of longer-lasting CFPS reactions and SCs for therapeutic and biomedical applications, as well as for reducing operational costs in industrial-scale protein production. Together, these advances position PS not only as an alternative energy donor, but as a tool for expanding when and how CFPS technologies can be applied.

## Materials and Methods

Detailed Materials and Methods are provided in the Supplementary.

### sfGFP In Vitro Production in CFPS

To assess the activity of a cell-free reaction, sfGFP fluorescence signal (measured at 488/530 nm) of samples prepared were measured using an Infinite 200 PRO multimode reader (TECAN, Austria) controlled by the i-control 1.10 software. For overtime assessment, the samples were measured in intervals of 10 min for 8 h, incubated at 30 °C. The point at which the GFP intensity derivative reaches zero indicates the cessation of protein production.

### Measurement of ATP Concentration in CFPS Reactions

To assess ATP concentrations in CFPS reactions over time, reactions containing a DNA template encoding sfGFP were incubated at 37 °C for various durations. At the end of each incubation period, GFP fluorescence was measured as described previously, and ATP concentrations were quantified using luminescence-based assay with the CellTiter-Glo 2.0 reagent (Promega, provided by IM Beit HaEmek, Israel).

### Energy Source Supplementation During CFPS Reaction

The sfGFP fluorescence signal of the cell-free reaction was measured over time, as described above, until sfGFP production plateaued. The supplementation volumes were calculated to achieve final concentrations of 1.2 mM ATP or 40 mM PGA. Samples that were not supplemented with an energy source received an equivalent volume of UPW. The initial concentrations of ATP and PGA in the reaction were 1.2 mM and 40 mM, respectively.

### The SCs Preparation Process

SCs preparation using the emulsion transfer method was performed as previously described.^67^ The preparation process is detailed in Supplementary Section 3.

### Statistical Analysis

The statistical analysis, including one-way and two-way analysis of variance (ANOVA), was performed using Prism GraphPad version 9.3.1 (GraphPad Software, Inc., La Jolla, CA). Statistical significance was set as *p = 0.0332 and ****p < 0.0001, with a 95% confidence interval.

## Supplementary Material



## Data Availability

All of the data needed to evaluate the conclusions in the paper are present in the paper and/or the Supporting Information. Correspondence and requests for materials should be addressed to AS (avids@technion.ac.il).

## Notes

CFPS reagent cost analysis; efficiency, affordability, and potency analyses; ATP, PGA, PS, serine, and glutamate supplementation experiments; chemical characterization of PGA and PS by ninhydrin assay, TLC, and ^1^H-NMR; LC-MS analysis of CFPS metabolites; proteomic analysis of glycolytic and serine biosynthesis enzymes; detailed materials and methods, including CFPS composition, lysate preparation, DNA vectors, protein sequences, enzymatic and immunoassay protocols, and SCs preparation and characterization (PDF)
