# Supplementary material for: Phosphoserine as an Alternative Energy Source for E. coli Cell-Free Protein Synthesis with Increased Yield and Prolonged Activity
Source: ACS Synth Biol. 2026 Jun 18;15(7):2823–36. doi: 10.1021/acssynbio.5c00649 (PMC13386643; doi:10.1021/acssynbio.5c00649)
Supplement: Supplementary file 1 [file sb5c00649_si_001.pdf]

Supporting Information for Publication

Phosphoserine as an Alternative Energy Source for *E. coli* Cell-Free Protein Synthesis with Increased Yield and Prolonged Activity

Shanny Ackerman[1], Yael Fink[1], Yasmin Habib[1], Devora Cohen-Karni[2], Omer Adir[3], Gal Chen[1], Yuval Richtman[1], Sónia Siquenique[4,5,6], Michael Levi[7], Jeny Shklover[1], Bruno Sarmento[4,5,8], Avi Schroeder[1*]

[1] The Louis Family Laboratory for Targeted Drug Delivery and Personalized Medicine Technologies, Department of Chemical Engineering, Technion – Israel Institute of Technology, Haifa, 32000, Israel.

[2] College of Osteopathic Medicine, Lake Erie College of Osteopathic Medicine, Greensburg, PA, 15601, USA.

[3] Department of Biological Engineering, MIT, Cambridge, MA, 02139, USA

[4] i3S – Instituto de Investigação e Inovação em Saúde, Universidade do Porto, Porto, 4099-002, Portugal

[5] INEB – Instituto de Engenharia Biomédica, Universidade do Porto, Porto, 4099-002, Portugal

[6] ICBAS–Instituto de Ciências Biomédicas Abel Salazar, Universidade do Porto, Porto, 4099-002, Portugal

[7] Department of Biotechnology and Food Engineering, Technion – Israel Institute of Technology, Haifa, 32000, Israel

[8] IUCS-CESPU – Instituto Universitário de Ciências da Saúde, Gandra, 4585-116, Portugal

* corresponding author, avids@technion.ac.il

Table S1: CFPS reagents prices per reaction

Reagent	Stock conc.	For 100ul reaction (ul)	Mw (g/mol)	1ml stock (g/ml)	Price per gram ($/g)	1ml stock price ($/ml)	Price per 100ul reaction ($)
**PGA (2021-2023)***	0.5M	8	230	0.115	510	**58.65**	**0.469**
**PGA (2024)** (LabSuit Shop, Israel)					1820	**209.3**	**1.674**
**Amino acids***	50mM	10	-	-	1140	11.40	0.114
**GTP***	50mM	2	523.18	0.026	1177	30.79	0.062
**UTP***	100mM	0.8	586.12	0.059	480	28.13	0.023
**ATP***	100mM	1.2	551	0.055	208	11.46	0.014
**Sucrose****	2M	10	342.3	0.685	0.28	0.192	0.002
**HEPES** KOH pH=8**	1M	5.5	238.3	0.238	0.48	0.114	0.0006
**PEG6000****	50% v/w	6		0.5	0.13	0.065	0.0004
**IPTG** (Ornat, Rehovot, Israel)	100mM	1	238.31	0.024	12.3	0.293	0.0003
**Potassium acetate****	1M	5	98.15	0.098	0.048	0.005	2E-05
**Ammonium acetate****	5.2M	3	77.08	0.401	0.049	0.020	5.85E-05
**Magnesium acetate****	1M	1.4	142.4	0.142	0.27	0.038	5.38E-05
			**Total price per 100ul reaction:**		2021-2023		0.684
					2024		1.890

* Was Purchased from Sigma-Aldrich, Rehovot, Israel

** Was Purchased from Technion Chemical Store

Table S2: The efficiency of CFPS reactions with different concentrations of PGA and PS

Reaction energy sources		Price per 100ul reaction ($)	Reaction activity	Efficiency (activity / cost)	Relative efficiency to baseline reaction
**PGA (mM)**	**PS (mM)**				
**0**	**20**	0.233	360	1548	52.2%
	**40**	0.250	801	3204	108.1%
	**60**	0.267	1121	4196	141.6%
	**80**	0.284	1224	4303	145.2%
**20**	**0**	1.052	566	538	18.2%
	**20**	1.070	3596	3362	113.4%
	**40**	1.087	6420	**5906**	**199.3%**
	**60**	1.104	6537	5919	199.7%
**40**	**0**	1.890	5600	2964	100.0%
	**20**	1.907	11377	5966	201.3%
	**40**	1.924	8726	4535	153.0%
	**60**	1.942	8478	4366	147.3%
**60**	**0**	2.727	12143	4453	150.3%
	**20**	2.744	9214	3358	113.3%
	**40**	2.761	6358	2302	77.7%
	**60**	2.779	4214	1516	51.2%

Gray background- Baseline reaction

XXX- the alternatives with the highest efficiency

XXX- the alternative with the highest efficiency at the lowest cost

Table S3: The affordability and potency of CFPS reactions with different concentrations of PGA and PS

Reaction energy sources		Price per 100ul reaction ($)	Reaction activity	Efficiency (activity / cost)	Affordability (efficiency / cost)	Relative affordability	Potency (efficiency X activity)	Relative potency
**PGA (mM)**	**PS (mM)**							
**0**	**20**	0.233	360	1548	6657	424.4%	5.57E+05	3.4%
	**40**	0.25	801	3204	12825	817.7%	2.57E+06	15.5%
	**60**	0.267	1121	4196	**15706**	**1001.4%**	4.70E+06	28.3%
	**80**	0.284	1224	4303	15124	964.3%	5.27E+06	31.7%
**20**	**0**	1.052	566	538	511	32.6%	3.05E+05	1.8%
	**20**	1.07	3596	3362	3143	200.4%	1.21E+07	72.8%
	**40**	1.087	6420	5906	5433	346.4%	3.79E+07	228.5%
	**60**	1.104	6537	5919	5360	341.7%	3.87E+07	233.1%
**40**	**0**	1.89	5600	2964	1568	100.0%	1.66E+07	100.0%
	**20**	1.907	11377	5966	3129	199.5%	**6.79E+07**	**409.0%**
	**40**	1.924	8726	4535	2357	150.3%	3.96E+07	238.4%
	**60**	1.942	8478	4366	2249	143.4%	3.70E+07	223.0%
**60**	**0**	2.727	12143	4453	1633	104.1%	5.41E+07	325.8%
	**20**	2.744	9214	3358	1224	78.0%	3.09E+07	186.4%
	**40**	2.761	6358	2302	834	53.2%	8.50E+04	88.4%
	**60**	2.779	4214	1516	546	34.8%	8.50E+04	38.5%

Gray background- Baseline reaction

XXX- the alternative with the lowest cost relative to its efficiency

XXX- the alternative with the highest activity relative to its efficiency

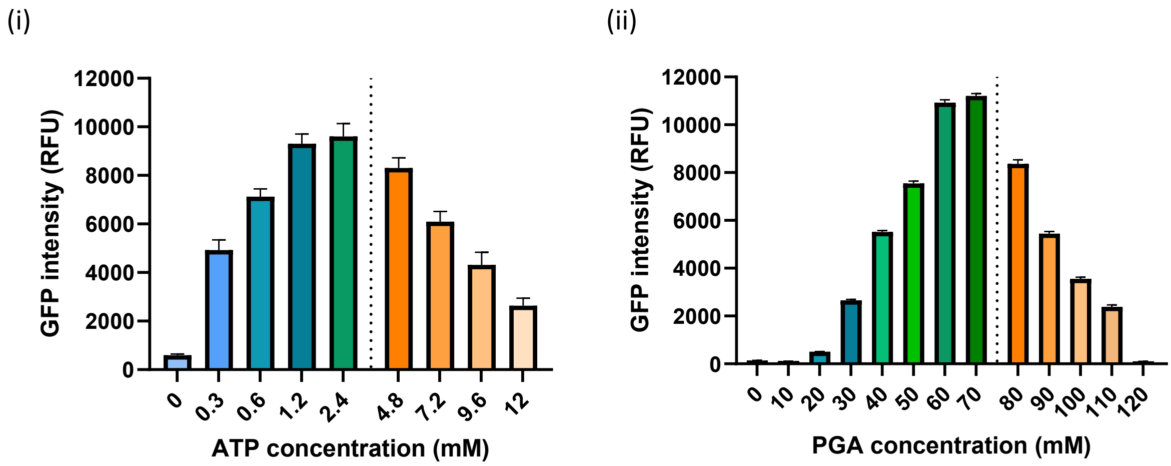

Figure S1: Superfolder green fluorescent protein (sfGFP) production over 8 hours in an *E. coli*-based CFPS reaction at 37°C with different initial concentrations of energy sources, (i) ATP and (ii) PGA. Data are expressed as mean ± s.e.m (n = 3 independent samples).

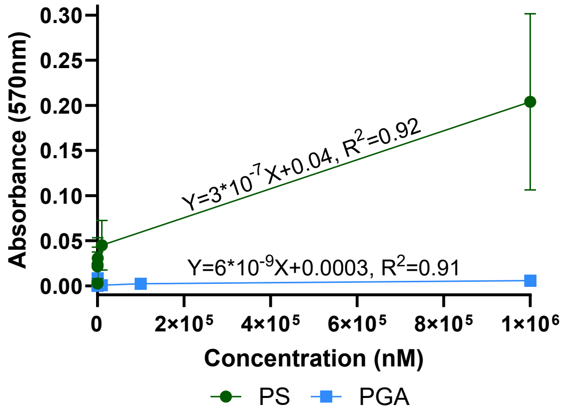

Figure S2: Standard curves of 3-phosphoglyceric acid (PGA) and phosphoserine (PS) concentrations ranging from 10nM to 1mM determined by colorimetric ninhydrin assay. Data are expressed as mean ± s.e.m (n=4 independent samples).

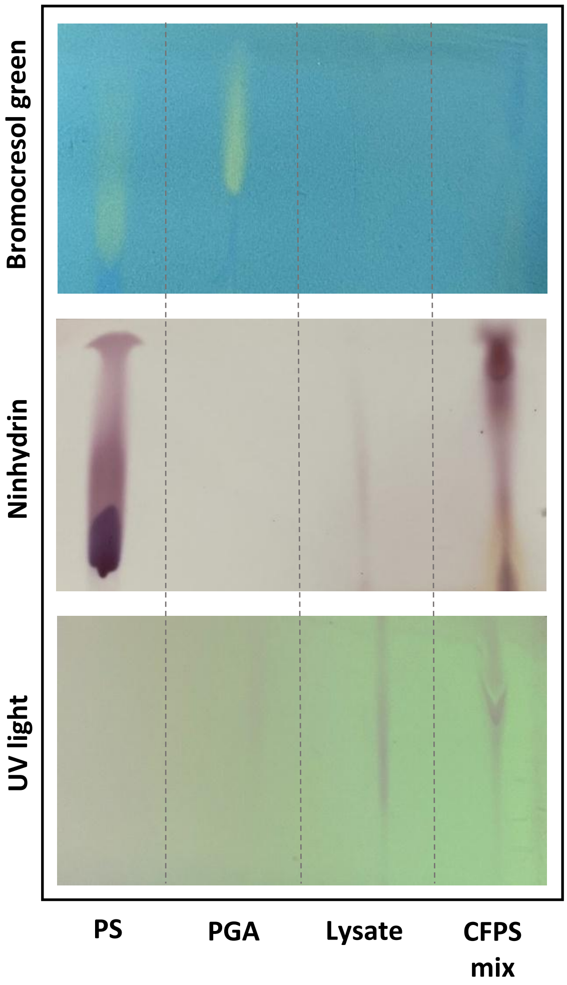

Figure S3: Thin-layer chromatography (TLC) of 1M PS solution, 1M PGA solution, bacterial lysate and cell-free protein synthesis mixture solution, visualized with bromocresol green stain solution, ninhydrin stain solution and UV light.

(i)

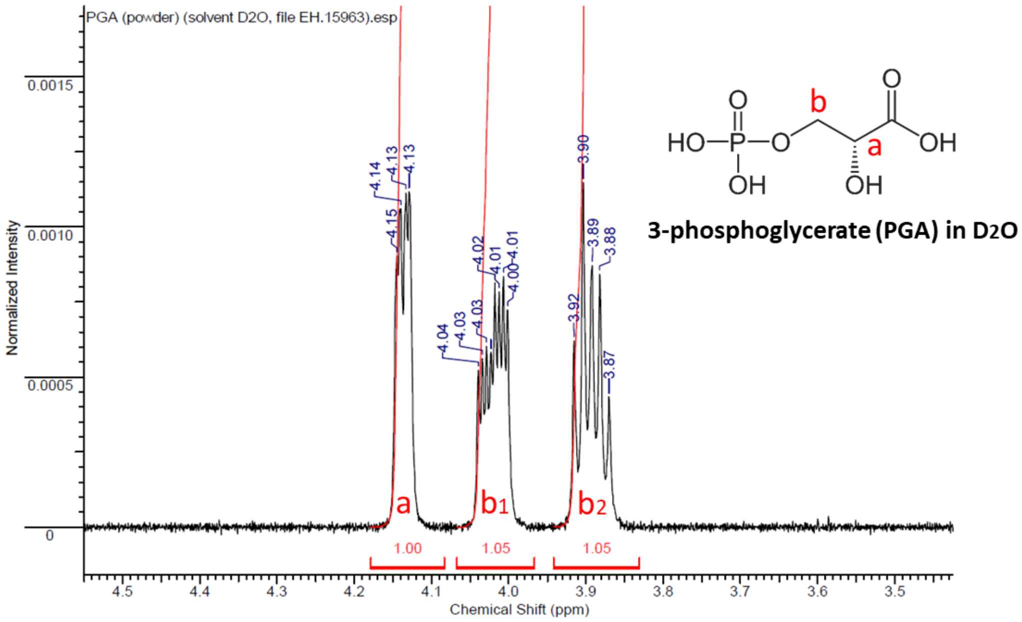

(ii)

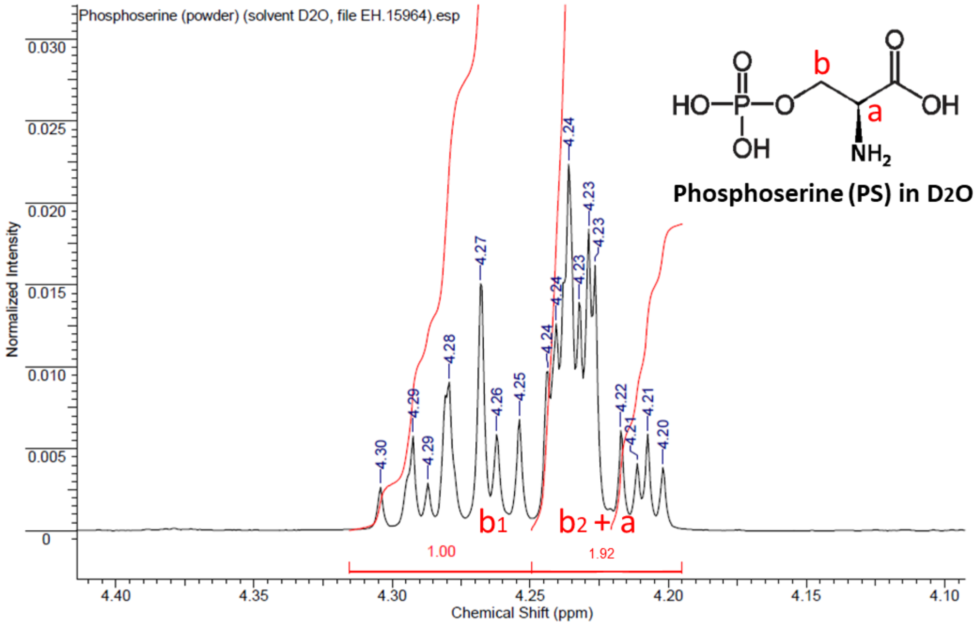

Figure S4: Nuclear magnetic resonance (^{1}H-NMR) spectrum of (i) PGA and (ii) PS in D_2O.

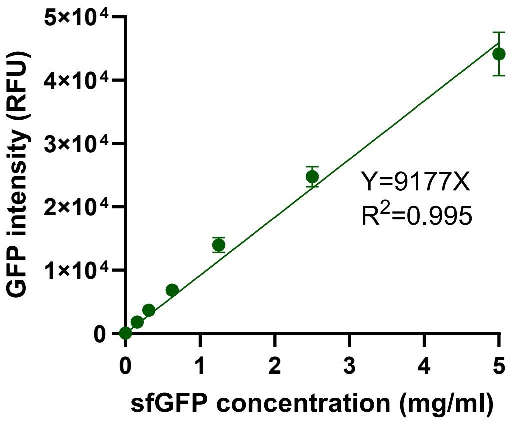

Figure S5: Calibration curve of sfGFP-purified protein mixed with non-producing cell free protein synthesis system (harboring the empty pET9a plasmid). Fluorescence intensity values were measured using a plate reader (n = 3).

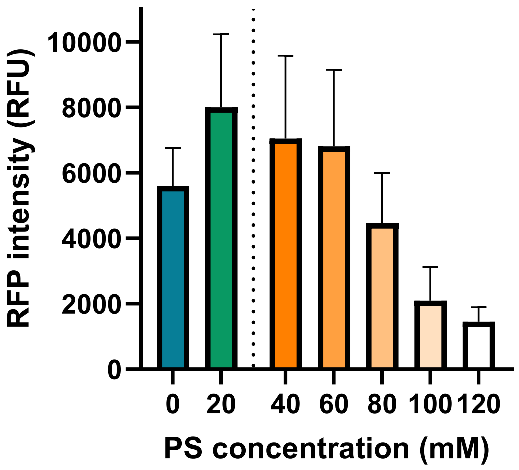

Figure S6: Monomeric red fluorescent protein 1 (mRFP1) production over 8 hours in an *E. coli*-based CFPS reaction at 37°C with 40mM PGA and different initial concentrations of PS. Data are expressed as mean ± s.e.m (n=6 independent samples).

(i) Glutamine

Phosphoserine ↔ 3-phosphohydroxypyruvate[1]

2-oxoglutarate **Glutamate**

Glutamate + Ammonia ↔ Glutamine[2-4]

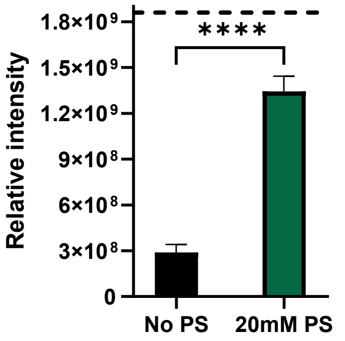

(ii) Pyruvate

Phosphoserine ↔ 3-phosphohydroxypyruvate ↔ **PGA** [1]

PGA ↔ 2-phosphoglycerate ↔ Phosphoenolpyruvate ↔ Pyruvate[5]

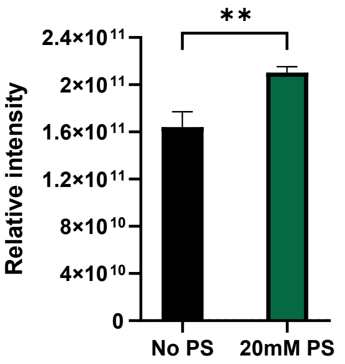

(iii) Glyceraldehyde 3-Phosphate

PGA ↔ 1,3-Bisphosphoglycerate ↔ Glyceraldehyde 3-Phosphate[5]

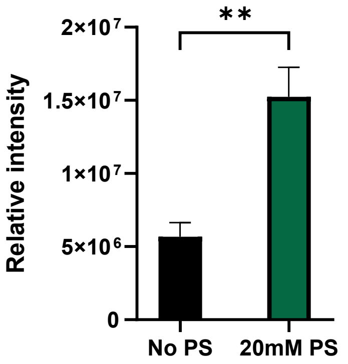

Figure S7: LC-MS analysis of relative levels of (i) Glutamine, (ii) Pyruvate, and (iii) Glyceraldehyde 3-Phosphate in CFPS reactions with and without 20 mM PS, measured shortly after mixing. Dashed lines indicate metabolite levels in reactions lacking *E. coli* lysate, corresponding to the initial concentrations added. Data are presented as mean ± s.e.m. Unpaired two-tailed t test P value, **P=0.0021, ****P=<0.0001 (n = 3 independent samples).

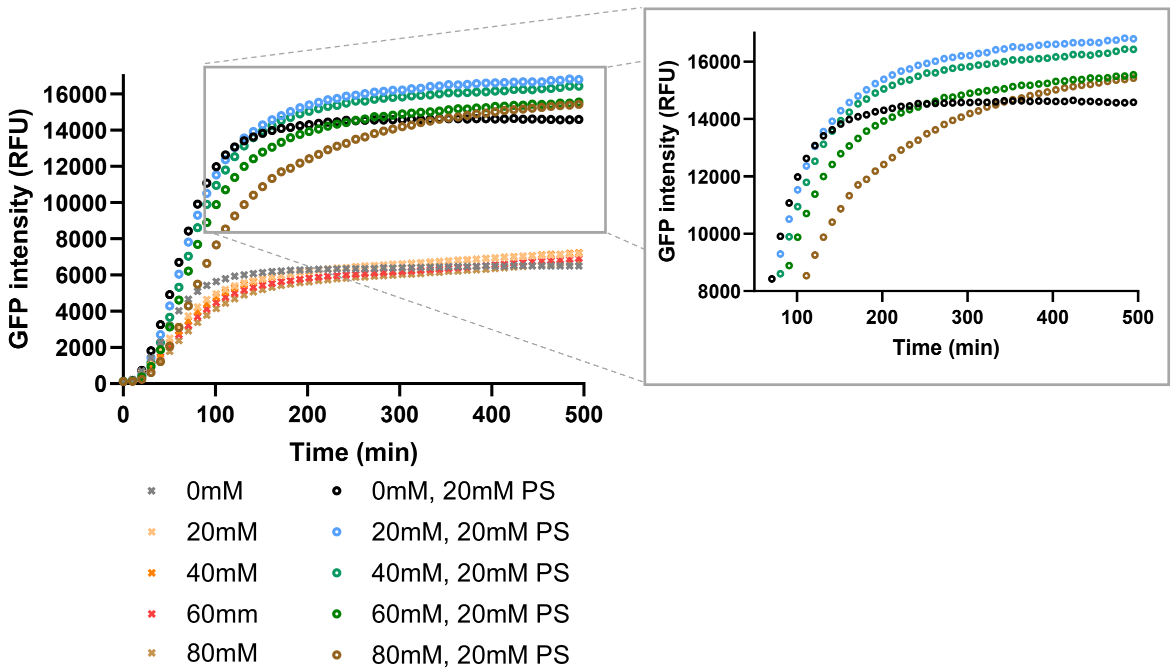

Figure S8: The effect of glutamate on CFPS activity. sfGFP production over 8 hours at 37°C in an *E. coli*-based CFPS reaction containing 40mM PGA, with or without 20mM PS, and with varying glutamate concentrations (n = 4 independent samples).

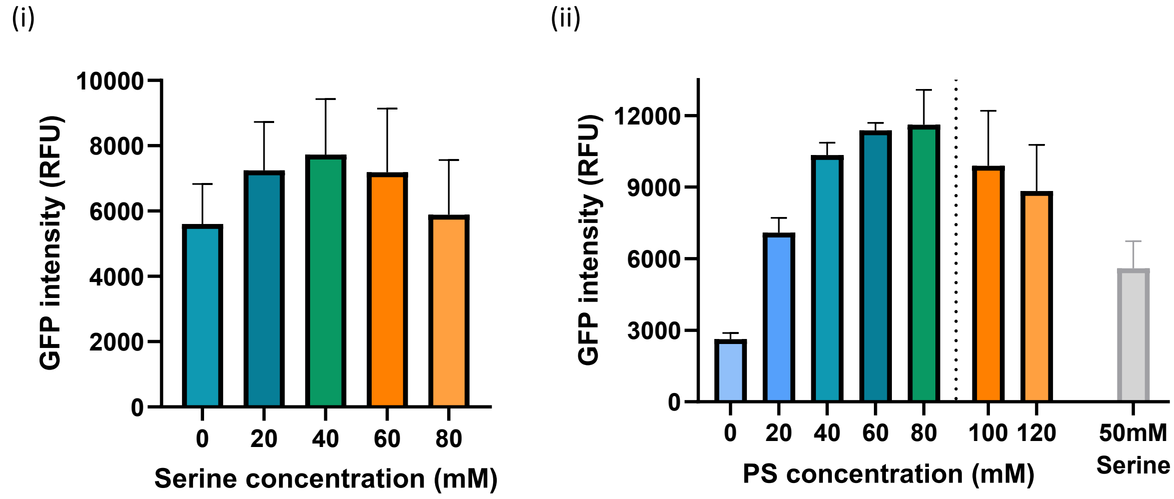

Figure S9: The effect of serine on CFPS activity. sfGFP production over 8 hours in an *E. coli*-based CFPS reaction containing 40mM PGA with varying serine concentrations: (i) increasing initial serine concentrations, in addition to the original 50mM serine present in the CFPS reaction; (ii) CFPS reactions without serine, supplemented with different PS concentrations. Data are expressed as mean ± s.e.m (n=6 independent samples).

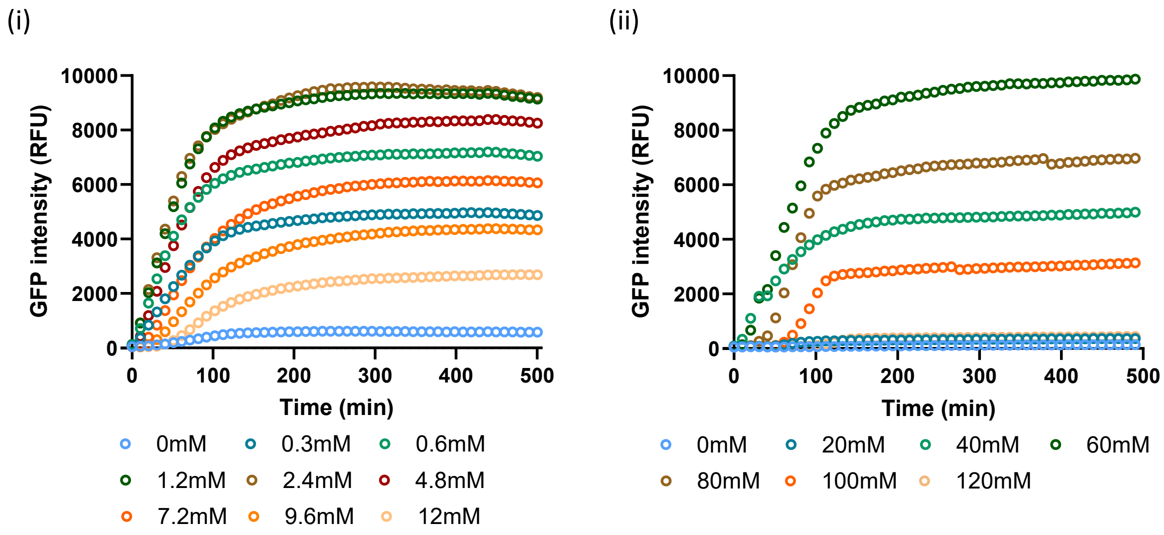

Figure S10: sfGFP production over time in a CFPS reaction at 37°C with different initial concentrations of (i) ATP and (ii) PGA (n = 3-5 independent samples).

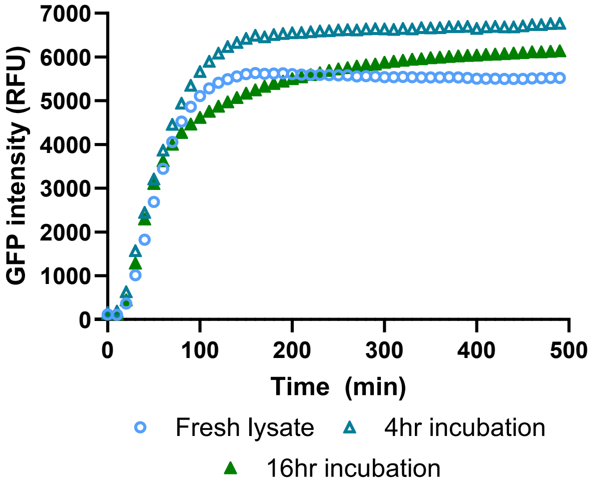

Figure S11: sfGFP production over time in a CFPS reaction at 37°C, using *E. coli* lysate that was pre-incubated at 37°C for various durations prior to the experiment (n = 3–5 independent samples).

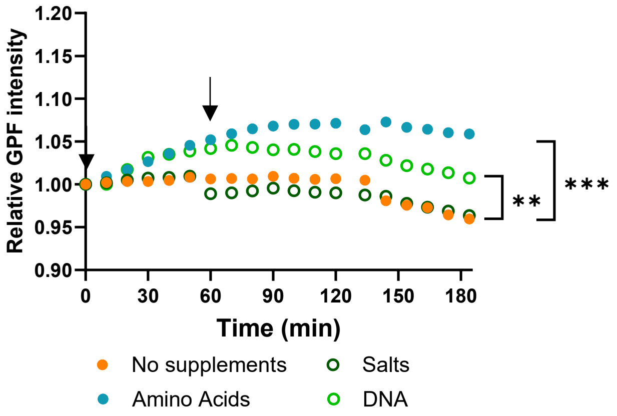

Figure S12: The effect of supplementation of CFPS reagents to CFPS reaction after sfGFP production plateaued. The addition of the reagents is marked by the arrows. All values are normalized to the amount of GFP in the solution at the time of the first addition. Time 0 indicates the first supplementation to the test samples after the original GPF production plateaued. Two-way ANOVA with adjusted P value in Tukey's multiple comparisons tests, *P=0.0332, **P=0.0021 (n = 3 independent samples).

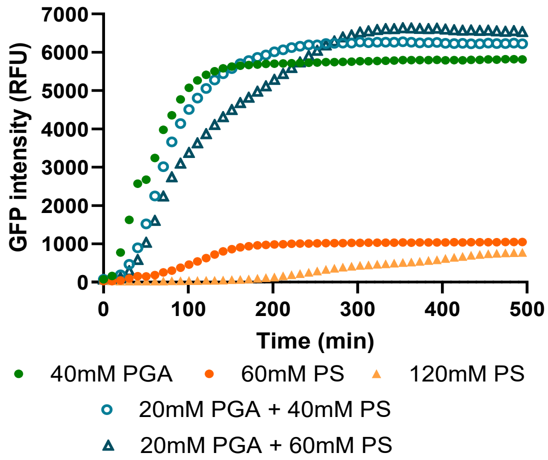

Figure S13: sfGFP production over-time in an CFPS reaction with different initial concentrations of PGA and PS (n = 3-6 independent samples).

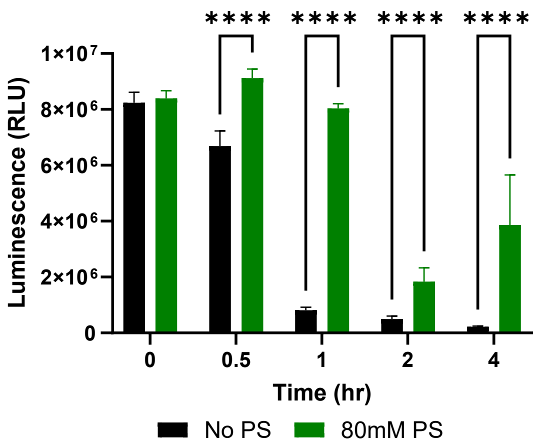

Figure S14: Comparison of ATP concentrations over time in CFPS reactions containing 40mM PGA with or without 80mM PS. Two-way ANOVA with adjusted P value in Šídák's multiple comparisons test, P=>0.1234, ****P =<0.0001 (n = 3 independent samples).

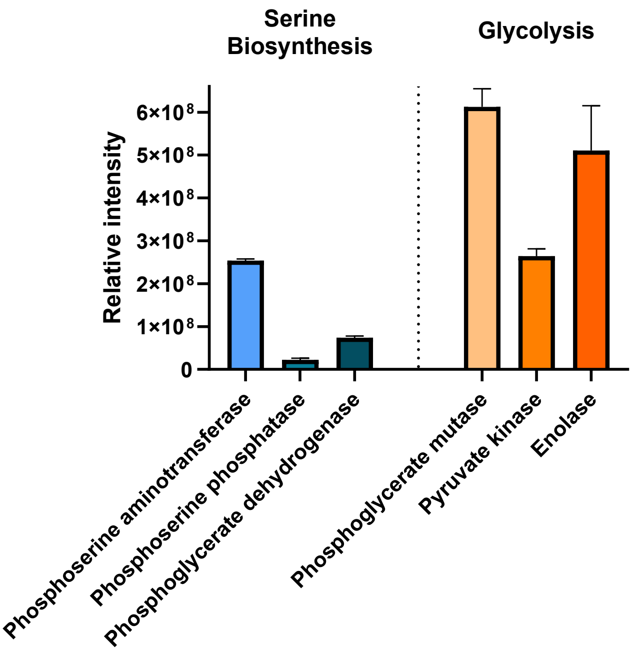

Figure S15: Relative intensities of glycolytic and serine biosynthesis enzymes in E. coli lysate measured by proteomic analysis (n = 3 independent samples).

Materials and methods:

1. **Cell-free protein synthesis (CFPS) composition**

1.1. S30-T7 Lysate preparation:

S30 bacterial lysate was prepared as previously described[6,7] from BL21(DE3) E. coli (New England Biolabs, USA) transformed with the T7 polymerase-expressing TargeTron vector pAR1219 (Sigma-Aldrich, Rehovot, Israel), with minor modifications: the bacterial growth volume was increased to 1500 ml, S30 buffer was prepared without 2-mercaptoethanol, and was enriched with 2 mM DTT instead of 1 mM DTT.

1.2. CFPS reaction

CFPS system reactions were performed as previously described[6,7], using in-house S30-T7 bacterial lysate (Table S4) . For different concentrations of ATP or PGA, or for the addition of phosphoserine (PS), serine or glutamate the UPW volume was adjusted.

Table S4: CFPS reaction composition

Reagent	Stock concentration	Final concentration
HEPES KOH pH=8	1 M	55 mM
Magnesium acetate	1 M	14 mM
Potassium acetate	1 M	50 mM
Ammonium acetate	5.2 M	155 mM
Polyethylene glycol (PEG) 6000	50% w/v	1.5% w/v
3-Phosphoglyceric acid (3-PGA)	0.5 M	40 mM
Amino acids - mixture I	50 M	2.5 mM
Amino acids - mixture II	50 M	2.5 mM
ATP	100 mM	1.2 mM
GTP	50 mM	1 mM
UTP	100 mM	0.8 mM
IPTG	100 mM	1 mM
Sucrose	2 M	200 mM
S30-T7 lysate		34% v/v
DNA plasmid		10 ng/µL
Ultrapure water (UPW)		To total reaction volume

* UPW should be added to complete the final required volume.

** Plasmid DNA volume addition according to stock concentration

1.3. 1M Phosphoserine (PS) solution:

Phosphoserine (Sigma-Aldrich, Rehovot, Israel) was weighted and dissolved in 2M KOH solution (in UPW) for final concentration of 1M.

1.4. 1M Glutamate solution:

L-glutamic acid (Sigma-Aldrich, Rehovot, Israel) was weighted and dissolved in 1.2M KOH solution (in UPW) for final concentration of 1M.

1.5. DNA vectors:

The DNA template encoding super-folder green fluorescent protein (sfGFP) was procured from Sandia BioTech (Albuquerque, New Mexico, USA). This DNA template was then cloned into a pET9a vector, incorporating a histidine×6 tag, as previously described in a study conducted by our group[1].

The DNA plasmid pBLind containing the gene for monomeric red fluorescent protein 1 (mRFP1) was procured from Addgene.

The DNA plasmid pCI-T7Max-UTR1-CTerminus8xHis-T500 containing the gene for Firefly luciferase (Fluc) was procured from Addgene.

A pET9d plasmid encoding Tyrosinase from *Bacillus megaterium* was kindly provided by Prof. Ayelet Fishman[8].

The DNA plasmid DNA encoding human wild-type α-synuclein (pT7-7 α-syn WT) was a kind gift from Hilal Lashuel (Addgene).

1.6. Protein sequences

Name	Sequence
sfGFP-6His	ATGAGCAAAGGAGAAGAACTTTTCACTGGAGTTGTCCCAATTCTTGTTGAATTAGATGGTGATG TTAATGGGCACAAATTTTCTGTCCGTGGAGAGGGTGAAGGTGATGCTACAAACGGAAAACTCA CCCTTAAATTTATTTGCACTACTGGAAAACTACCTGTTCCATGGCCAACACTTGTCACTACTCTG ACCTATGGTGTTCAATGCTTTTCCCGTTATCCGGATCACATGAAACGGCATGACTTTTTCAAGAG TGCCATGCCCGAAGGTTATGTACAGGAACGCACTATATCTTTCAAAGATGACGGGACCTACAA GACGCGTGCTGAAGTCAAGTTTGAAGGTGATACCCTTGTTAATCGTATCGAGTTAAAAGGTATT GATTTTAAAGAAGATGGAAACATTCTCGGACACAAACTCGAGTACAACTTTAACTCACACAATG TATACATCACGGCAGACAAACAAAAGAATGGAATCAAAGCTAACTTCAAAATTCGCCACAACG TTGAAGATGGTTCCGTTCAACTAGCAGACCATTATCAACAAAATACTCCAATTGGCGATGGCCC TGTCCTTTTACCAGACAACCATTACCTGTCGACACAATCTGTCCTTTCGAAAGATCCCAACGAAA AGCGTGACCACATGGTCCTTCTTGAGTTTGTAACTGCTGCTGGGATTACACATGGCATGGATGA GCTCTACAAAGGATC
mRFP1	ATGGCGAGTAGCGAAGACGTTATCAAAGAGTTCATGCGTTTCAAAGTTCGTATGGAAGGTTCC GTTAACGGTCACGAGTTCGAAATCGAAGGTGAAGGTGAAGGTCGTCCGTACGAAGGTACCCA GACCGCTAAACTGAAAGTTACCAAAGGTGGTCCGCTGCCGTTCGCTTGGGACATCCTGTCCCCG CAGTTCCAGTACGGTTCCAAAGCTTACGTTAAACACCCGGCTGACATCCCGGACTACCTGAAAC TGTCCTTCCCGGAAGGTTTCAAATGGGAACGTGTTATGAACTTCGAAGACGGTGGTGTTGTTAC CGTTACCCAGGACTCCTCCCTGCAAGACGGTGAGTTCATCTACAAAGTTAAACTGCGTGGTACC AACTTCCCGTCCGACGGTCCGGTTATGCAGAAAAAAACCATGGGTTGGGAAGCTTCCACCGAA CGTATGTACCCGGAAGACGGTGCTCTGAAAGGTGAAATCAAAATGCGTCTGAAACTGAAAGAC GGTGGTCACTACGACGCTGAAGTTAAAACCACCTACATGGCTAAAAAACCGGTTCAGCTGCCG GGTGCTTACAAAACCGACATCAAACTGGACATCACCTCCCACAACGAAGACTACACCATCGTTG AACAGTACGAACGTGCTGAAGGTCGTCACTCCACCGGTGCTTAA
Fluc	ATGGAAGACGCCAAAAACATAAAGAAAGGCCCGGCGCCATTCTATCCGCTGGAAGATGGAAC CGCTGGAGAGCAACTGCATAAGGCTATGAAGAGATACGCCCTGGTTCCTGGAACAATTGCTTT TACAGATGCACATATCGAGGTGGACATCACTTACGCTGAGTACTTCGAAATGTCCGTTCGGTTG GCAGAAGCTATGAAACGATATGGGCTGAATACAAATCACAGAATCGTCGTATGCAGTGAAAAC TCTCTTCAATTCTTTATGCCGGTGTTGGGCGCGTTATTTATCGGAGTTGCAGTTGCGCCCGCGA ACGACATTTATAATGAACGTGAATTGCTCAACAGTATGGGCATTTCGCAGCCTACCGTGGTGTT CGTTTCCAAAAAGGGGTTGCAAAAAATTTTGAACGTGCAAAAAAAGCTCCCAATCATCCAAAA AATTATTATCATGGATTCTAAAACGGATTACCAGGGATTTCAGTCGATGTACACGTTCGTCACA TCTCATCTACCTCCCGGTTTTAATGAATACGATTTTGTGCCAGAGTCCTTCGATAGGGACAAGAC AATTGCACTGATCATGAACTCCTCTGGATCTACTGGTCTGCCTAAAGGTGTCGCTCTGCCTCATA GAACTGCCTGCGTGAGATTCTCGCATGCCAGAGATCCTATTTTTGGCAATCAAATCATTCCGGA TACTGCGATTTTAAGTGTTGTTCCATTCCATCACGGTTTTGGAATGTTTACTACACTCGGATATTT GATATGTGGATTTCGAGTCGTCTTAATGTATAGATTTGAAGAAGAGCTGTTTCTGAGGAGCCTT CAGGATTACAAGATTCAAAGTGCGCTGCTGGTGCCAACCCTATTCTCCTTCTTCGCCAAAAGCA CTCTGATTGACAAATACGATTTATCTAATTTACACGAAATTGCTTCTGGTGGCGCTCCCCTCTCT AAGGAAGTCGGGGAAGCGGTTGCCAAGAGGTTCCATCTGCCAGGTATCAGGCAAGGATATGG GCTCACTGAGACTACATCAGCTATTCTGATTACACCCGAGGGGGATGATAAACCGGGCGCGGT

	CGGTAAAGTTGTTCCATTTTTTGAAGCGAAGGTTGTGGATCTGGATACCGGGAAAACGCTGGG<br>CGTTAATCAAAGAGGCGAACTGTGTGTGAGAGGTCCTATGATTATGTCCGGTTATGTAAACAA<br>TCCGGAAGCGACCAACGCCTTGATTGACAAGGATGGATGGCTACATTCTGGAGACATAGCTTA<br>CTGGGACGAAGACGAACACTTCTTCATCGTTGACCGCCTGAAGTCTCTGATTAAGTACAAAGGC<br>TATCAGGTGGCTCCCGCTGAATTGGAATCCATCTTGCTCCAACACCCCAACATCTTCGACGCAG<br>GTGTCGCAGGTCTTCCCGACGATGACGCCGGTGAACTTCCCGCCGCCGTTGTTGTTTTGGAGCA<br>CGGAAAGACGATGACGGAAAAAGAGATCGTGGATTACGTCGCCAGTCAAGTAACAACCGCGA<br>AAAAGTTGCGCGGAGGAGTTGTGTTTGTGGACGAAGTACCGAAAGGTCTTACCGGAAAACTC<br>GACGCAAGAAAAATCAGAGAGATCCTCATAAAGGCCAAGAAGGGCGGAAAGATCGCCGTG
Tyrosinase	ATGGGTAACAAGTATAGAGTTAGAAAAAACGTATTACATCTTACCGACACGGAAAAAAG<br>AGATTTTGTTCGTACCGTGCTAATACTAAAGGAAAAAGGGATATATGACCGCTATATAGC<br>CTGGCATGGTGCAGCAGGTAAATTTCATACTCCTCCGGGCAGCGATCGAAATGCAGCA<br>CATATGAGTTCTGCTTTTTTACCGTGGCATCGTGAATACCTTTTACGATTCGAACGTGAC<br>CTTCAGTCAATCAATCCAGAAGTAACCCTTCCTTATTGGGAATGGGAAACGGACGCACA<br>GATGCAGGATCCCTCACAATCACAAATTTGGAGTGCAGATTTTATGGGAGGAAACGGAA<br>ATCCCATAAAAGATTTTATCGTCGATACCGGGCCATTTGCAGCTGGGCGCTGGACGAC<br>GATCGATGAACAAGGAAATCCTTCCGGAGGGCTAAAACGTAATTTTGGAGCAACGAAA<br>GAGGCACCTACACTCCCTACTCGAGATGATGTCCTCAATGCTTTAAAAATAACTCAGTAT<br>GATACGCCGCCTTGGGATATGACCAGCCAAAACAGCTTTCGTAATCAGCTTGAAGGATT<br>TATTAACGGGCCACAGCTTCACAATCGCGTACACCGTTGGGTTGGCGGACAGATGGGC<br>GTTGTGCCTACTGCTCCGAATGATCCTGTCTTCTTTTTACACCACGCAAATGTGGATCG<br>TATTTGGGCTGTATGGCAAATTATTCATCGTAATCAAAACTATCAGCCGATGAAAAACGG<br>GCCATTTGGTCAAAACTTTAGAGATCCGATGTACCCTTGGAATACAACCCCTGAAGACG<br>TTATGAACCATCGAAAGCTTGGGTACGTATACGATATAGAATTAAGAAAATCAAAACGTT<br>CCTCATAA

1.7. Fluc in-vitro production in CFPS

CFPS reactions were supplemented with 10 ng/µL DNA encoding Fluc and incubated at 30°C. At designated time points, aliquots were removed for luminescence analysis. For measurement, reaction mixtures were prepared at a volume ratio of 1:18:1 (CFPS reaction : 1M HEPES buffer : OneGlo Luciferase Assay Reagent (Promega, provided by IM Beit HaEmek, Israel)) and incubated for 10 min in room temperature. Luminescence was measured using an Infinite 200 PRO multimode reader.

1.8. Tyrosinase in-vitro production in CFPS

CFPS reactions were supplemented with 10 ng/µL DNA encoding Tyrosinase and incubated at 37°C with shaking at 1200rpm. Control reactions lacking DNA were prepared in parallel. At each designated time point, reaction tubes were centrifuged at 20,000g for 10 min at 4°C and the supernatants were used immediately for enzymatic activity measurements.

1.9. L-DOPA assay

Tyrosinase activity was determined using a L-DOPA oxidation assay. Reaction mixtures were prepared at a volume ratio of 4:3:1:1 (supernatant : 0.5M PS in 1M KOH : 10mM $CuSO_4$: 5mM L-DOPA). L-DOPA stock solution was prepared in advance and kept on ice until use. Absorbance at 475nm was monitored after 90 min at 37°C using an Infinite 200 PRO multimode reader.

1.10. α-synuclein in-vitro production in CFPS

CFPS reactions were supplemented with 10 ng/uL DNA encoding α-synuclein and incubated at 37°C with shaking of 1200 rpm. At each designated time point, reaction were heated to 80°C for 5 minutes to stop the reaction and then centrifuge at 20,000g for 10 minutes at 4°C. The supernatants were stored at -20°C before preforming the ELISA.

1.11. Enzyme-Linked Immunosorbent Assay (ELISA) to detect α-synuclein

An indirect ELISA was performed to detect α-synuclein protein. High-binding 96-well plates were coated with defrosted samples diluted in coating buffer and incubated overnight at 4°C. The next day, plates were washed with PBST (PBS with 0.05% Tween-20) and blocked with 1% BSA in PBST for 1 hour at room temperature. The wells were then incubated with the primary antibody MJFR1 (Abcam, ab138501, a monoclonal antibody specific for α-synuclein) diluted 1:1000 in blocking buffer, for 1.5 hours at room temperature, washed with PBST, and subsequently incubated with an HRP-conjugated secondary antibody diluted 1:10,000 in blocking buffer, for additional 1.5 hours at room temperature. TMB ELISA Substrate (ab171522; Abcam) was used for signal development, and the absorbance at 650nm was monitored for 60 min with 2 min intervals at 37°C using an Infinite 200 PRO multimode reader.

1.12. Proteomics of the lysate batches

CFPS reactions, with and without 20 mM PS, were prepared. At the indicated time points (0, 1, and 8 hours of protein expression), reactions were rapidly frozen in liquid nitrogen to terminate activity and stored at -80°C.

The protein samples were brought to 8.5M Urea, 100mM ammonium bicarbonate and 10mM DTT. Protein amount was estimated using Bradford readings. The samples were reduced (60°C for 30 min), modified with 35.2mM iodoacetamide in 100mM ammonium bicarbonate (room temperature for 30 min in the dark) and digested in 1.5M Urea, 17.6mM ammonium bicarbonate with modified trypsin (Promega), overnight at 37°C in a 1:50 (M/M) enzyme-to-substrate ratio. An additional second digestion with Trypsin was done for 4 hours at 37°C in a 1:100 (M/M) enzyme-to-substrate ratio. The tryptic peptides were desalted using Oasis HLB 96-well µElution Plate (Waters), dried and re-suspended in 0.1% Formic acid in 2% acetonitrile.

The resulting peptides were analyzed by LC-MS/MS using an Exploris 480 mass spectrometer (Thermo) fitted with a capillary UHPLC (Vanquish Neo, Thermo scientific). The peptides were loaded in solvent A (0.1% formic acid in water) on an C18 reversed phase analytical column (Ionoptics, AUR3-25075C18-XT, 25cm x 75um ID, 1.7um). The peptides mixture was resolved with a 6 to 34% linear gradient of solvent B (80% acetonitrile with 0.1% formic acid in water) for 120 minutes followed by gradient of 0.1 min increase of 34 to 99% and 14 minutes at 99% solvent B at flow rates of 0.15 µl/min. Mass spectrometry was performed in a positive mode using repetitively full MS scan (m/z 380–985, resolution 120,000) followed by DIA scans (10Da isolation windows with 1 m/z overlap, and resolution 30,000).

The mass spectrometry data was analyzed using the DIA-NN software version 2.2.0[9,10] searching against the e. coli bl21 proteome from the Uniprot database, with minimal peptide length set to 7, maximum number of missed cleavages set to 1, cysteine carbamidomethylation enabled as a fixed modification, and protein N-term acetylation and oxidation on methionine enabled as variable modifications.

Peptide- and protein-level false discovery rates (FDRs) were filtered to 1%.

Combining the proteins and peptide files was done using Perseus 1.6.7 software[11].

2. Chemical analysis of PGA and PS

2.1. Ninhydrin assay

The ninhydrin assay was preformed according to Li at el[12] with slight modifications. Briefly, 30ml of ninhydrin solution was prepared by mixing 250mg of ninhydrin (Holland Moran, Yehud-Monosson, Israel), 7.5mg of ascorbic acid (Sigma-Aldrich, Rehovot, Israel) and 30ml of ethylene glycol (Sigma-Aldrich, Rehovot, Israel), followed by heating the mixture to 60°C for 1 hour. Additionally,100ml of 1M citrate buffer was prepared by dissolving 3.358gr of citric acid (Sigma-Aldrich, Rehovot, Israel) and 24.269gr of sodium citrate dihydrate (Sigma-Aldrich, Rehovot, Israel) in 100ml of distilled water, and adjusting the pH to pH=6 with 12M NaOH. The samples were mixed with the ninhydrin solution and

the citrate buffer in a 1:1:1 ratio and heated to 100◦C for 15 minutes. After additional 10 minutes at room temperature, the samples were vortexed vigorously, and their absorbance at 570nm was measured using a plate reader (Infinite 200 PRO multimode reader, TECAN, Austria controlled by the i-control 1.10 software).

2.2. Thin-layer chromatography (TLC)

A silica-gel TLC plate (Sigma-Aldrich, Rehovot, Israel) was cut to the desired size. On the application line, designated application points were marked with approximately 1.5cm spacing between them, and 1µL of each sample was spotted. After drying the solvent with a hair dryer, the TLC plate was placed in a glass beaker containing 24 mL of the mobile phase (1.5mL distilled water, 6.25mL methanol (Bio-Lab, Jerusalem, Israel), and 16.25mL chloroform (Bio-Lab, Jerusalem, Israel). The plate was allowed to develop until the solvent front reached the desired height. It was then removed from the beaker and dried with a hair dryer in a chemical hood. The plates were observed under UV light before being dipped in stain solutions. The ninhydrin stain solution was prepared by dissolving 1g of ninhydrin in 100mL of n-butanol (Sigma-Aldrich, Rehovot, Israel), followed by the addition of 3mL of acetic acid (Bio-Lab, Jerusalem, Israel). The bromocresol green stain solution was prepared by dissolving 40mg of bromocresol green (Sigma-Aldrich, Rehovot, Israel) in 100mL of absolute ethanol (Bio-Lab, Jerusalem, Israel), with 0.1M NaOH (Bio-Lab, Jerusalem, Israel) added dropwise until a blue color appeared. The stain solutions were stored at room temperature. For ninhydrin staining, the plate was dried on a preheated hot plate, while for bromocresol green staining, it was left to dry at room temperature

2.3. Nuclear magnetic resonance (NMR)

^{1}H-NMR analysis was performed by external supplier (IMI TAMI Institute for R&D Ltd) using D2O as the solvent. All the samples were analyzed under consistent experimental conditions.

3. SCs preparation

3.1. SCs' Lipids

1-palmitoyl-2-oleoyl-sn-glycero-3-phosphocholine (POPC) was purchased from Lipoid (Ludwigshafen, Germany). Cholesterol was purchased from Sigma-Aldrich (Rehovot, Israel). Rhodamine-labeled phospholipid-1,2-dimyristoyl-sn-glycero-3-phosphoethanolamine-N-(lissamine rhodamine B sulfonyl) (ammonium salt) (14:0 Liss Rhod PE) was purchased from (Avanti Lipids Polar, Alabaster, AL).

3.2. Preparation of lipid phase for SCs construction

POPC was lyophilized (FreeZone 2.5; Labconco, USA) overnight. POPC and cholesterol were dissolved separately in chloroform (Bio-Lab, Jerusalem, Israel) at a concentration of 80 mg/ml each, and then vortexed thoroughly. Subsequently, mineral oil (Sigma-Aldrich, Rehovot, Israel) was added to each solution to achieve a final lipid concentration of 20 mg/ml each. Each solution was vortexed, divided into 1 ml aliquots in Eppendorf vials, and then heated at 80°C and 450 RPM for one hour to evaporate the chloroform. The obtained POPC-oil and cholesterol-oil solutions' aliquots were stored at -20°C. For the preparation of the lipid-oil phase for the SCs process, both lipid-oil solutions were heated for 5 minutes at 37°C and vortexed before use. Then, the solutions were mixed at a 1:1 v/v ratio in the required amount (1:2 v/v ratio of inner solution to lipids in oil mixture). Rhodamine-labeled phospholipid (14:0 Liss Rhod PE, 1 mg/ml in ethanol) was incorporated by adding 0.4 µL to every 100 µL of lipid solution in mineral oil.

3.3. SCs inner reaction composition based on S30-T7 lysate and the corresponding Feeding solution

The CFPS inner solution, and the feeding solution were prepared as previously described[6,7,13] (Table S5).

Table S5: SC solution composition

Reagent	Stock concentration	Inner solution- final concentration	Feeding solution - final concentration
HEPES KOH pH=8	1 M	55 mM	83 mM
Magnesium acetate	1 M	14 mM	21 mM
Potassium acetate	1 M	50 mM	76 mM
Ammonium acetate	5.2 M	155 mM	236.4 mM
Polyethylene glycol (PEG) 6000	50% w/v	1.5% w/v	2.25% w/v
3-Phosphoglyceric acid (3-PGA)	0.5 M	40 mM	61 mM
Amino acids - mixture I	50 M	2.5 mM	3.8 mM
Amino acids - mixture II	50 M	2.5 mM	3.8 mM
ATP	100 mM	1.2 mM	1.8 mM
GTP	50 mM	1 mM	1.5 mM
UTP	100 mM	0.8 mM	1.2 mM
IPTG	100 mM	1 mM	1.5 mM
Sucrose	2 M	200 mM	-
Glucose	2 M	-	303 mM
S30-T7 lysate		34% v/v	-
DNA plasmid		10 ng/µL	-
Ultrapure water (UPW)		To total reaction volume	18.3 % (v/v)

* UPW should be added to complete the final required volume.

** plasmid DNA volume addition according to stock concentration

3.4. SCs preparation process

SCs were prepared using the water-in-oil emulsion transfer method as previously described[13]. Briefly, CFPS micelles were prepared by mixing 100µL of the inner solution with 200µL of the lipid phase in a 1.5mL plastic tube. The mixture was vigorously pipetted and moderately vortexed for 30 seconds to form an emulsion. The vial was then placed on ice for 10 minutes to allow emulsion stabilization. Next, the emulsion was gently dripped onto a 200mM glucose solution and centrifuged at 100g for 10 minutes at 4°C, followed by an additional centrifugation at 400g for 10 minutes at 4°C. The resulting particle pellet was transferred to a new 1.5mL tube and centrifuged again at 1000g for 10 minutes at 4°C. The supernatant was removed, and the pellet was resuspended in 100µL feeding solution. Protein production was carried out by incubating the sample at 37°C for 2 hours.

4. SCs Characterization

4.1. Fluorescent Microscopy Analysis of SCs

The Lionheart FX automated microscope (BioTek, Santa Clara, CA), controlled by Agilent Gen5 software (version 3.11), was used to visualize and analyze sfGFP-producing SCs, membrane-labeled with Rhodamine. The fabricated SCs were evaluated as follows: 5 µL of the SCs solution, either pre or post protein expression, was placed on a BSA-covered slide, and a cover slide was placed on top. For GFP-producing SCs, acquisition was performed using GFP and RFP filter cubes (EX 469/35 EM 525/39 and EX 531/40 EM 593/40, respectively) and LED cubes (465 nm and 523 nm, respectively), alongside brightfield visualization using 20X and 40X objectives.

4.2. Imaging flow cytometry analysis (size, concentration, activity, and percentage of active SCs)

The ImageStream®X Mk II (Luminex Corporation, USA), controlled by the AMNIS Inspire software (version 200.1.681.0) and analyzed using the IDEAS software (version 6.2), was used for this study. SCs, membrane-labeled with rhodamine, with plasmid DNA encoding for sfGFP, were incubated for 2

hours at 37°C and filtered with a 70µm cell strainer (BD Biosciences, San Jose, CA) to eliminate aggregates before analysis with the ImageStream®X. The 488 nm, 561 nm, and 785 nm lasers were used for fluorescence excitation along with side scatter (SSC) illumination (emission band 745-780 nm). SCs events were classified using a convolutional neural network as a model for a 3-class classification: active synthetic cells, inactive synthetic cells, and oil droplets, as previously described[13].

References:

1 Wulfert, S. & Krueger, S. Phosphoserine aminotransferase1 is part of the phosphorylated pathways for serine biosynthesis and essential for light and sugar-dependent growth promotion. *Frontiers in plant science* **9**, 1712 (2018).

2 Chiang, C.-J., Hu, M.-C., Ta, T. & Chao, Y.-P. Glutamate as a non-conventional substrate for high production of the recombinant protein in Escherichia coli. *Frontiers in Microbiology* **Volume 13 - 2022** (2022). https://doi.org/10.3389/fmicb.2022.991963

3 Helling, R. B. Pathway choice in glutamate synthesis in Escherichia coli. *J Bacteriol* **180**, 4571-4575 (1998). https://doi.org/10.1128/jb.180.17.4571-4575.1998

4 Ginsburg, A. in *Advances in Protein Chemistry* Vol. 26 (eds C. B. Anfinsen, John T. Edsall, & Frederic M. Richards) 1-79 (Academic Press, 1972).

5 Chandel, N. S. Glycolysis. *Cold Spring Harbor Perspectives in Biology* **13**, a040535 (2021).

6 Krinsky, N. *et al.* A simple and rapid method for preparing a cell-free bacterial lysate for protein synthesis. *PLoS One* **11**, e0165137 (2016).

7 Adir, O. *et al.* Preparing protein producing synthetic cells using cell free bacterial extracts, liposomes and emulsion transfer. *JoVE (Journal of Visualized Experiments)*, e60829 (2020).

8 Shuster, V. & Fishman, A. Isolation, Cloning and Characterization of a Tyrosinase with Improved Activity in Organic Solvents from Bacillus megaterium. *Journal of Molecular Microbiology and Biotechnology* **17**, 188-200 (2009). https://doi.org/10.1159/000233506

9 Messner, C. B. *et al.* Ultra-fast proteomics with Scanning SWATH. *Nature Biotechnology* **39**, 846-854 (2021). https://doi.org/10.1038/s41587-021-00860-4

10 Demichev, V., Messner, C. B., Vernardis, S. I., Lilley, K. S. & Ralser, M. DIA-NN: neural networks and interference correction enable deep proteome coverage in high throughput. *Nature Methods* **17**, 41-44 (2020). https://doi.org/10.1038/s41592-019-0638-x

11 Tyanova, S. *et al.* The Perseus computational platform for comprehensive analysis of (prote)omics data. *Nature Methods* **13**, 731-740 (2016). https://doi.org/10.1038/nmeth.3901

12 Li, Z., Chang, S., Lin, L., Li, Y. & An, Q. A colorimetric assay of 1-aminocyclopropane-1-carboxylate (ACC) based on ninhydrin reaction for rapid screening of bacteria containing ACC deaminase. *Letters in Applied Microbiology* **53**, 178-185 (2011). https://doi.org/https://doi.org/10.1111/j.1472-765X.2011.03088.x

13 Sharf-Pauker, N. *et al.* Scaling Up Synthetic Cell Production Using Robotics and Machine Learning Toward Therapeutic Applications. *Advanced Biology* **9**, 2400671 (2025). https://doi.org/https://doi.org/10.1002/adbi.202400671
